# Social support in schools and related outcomes for LGBTQ youth: a scoping review

**DOI:** 10.1007/s44217-022-00016-9

**Published:** 2022-11-14

**Authors:** Enoch Leung, Gabriela Kassel-Gomez, Samantha Sullivan, Flavio Murahara, Tara Flanagan

**Affiliations:** Department of Educational and Counseling Psychology, Faculty of Education, McGill University, Montreal, Canada

**Keywords:** Change, LGBTQ, Schools, Social support, Systems, Youth

## Abstract

Recent research has increasingly focused on positive factors and supports for LGBTQ youth. This scoping review explores existing social support for LGBTQ youth in schools through the Ecological Systems Theory to respond to the following four objectives: (1) define social support systems in schools, (2) identify current research on outcomes for LGBTQ youth, (3) identify barriers to support LGBTQ youth in schools, and (4) identify areas for future research for LGBTQ youth and social support in schools. A systematic search (Arksey and O’Malley in Int J Soc Res Methodol 8(1):19–32, 2005) between 2007 through 2021 resulted in 94 articles. This review gave rise to an organizational framework to consolidate various systems of social support for LGBTQ youth in schools. Social support consisted of seven social support systems (family, curriculum, family, peers, school policies, GSAs and programs, and school climate) that are positively associated with the promotion of positive socioemotional, behavioural, and educational outcomes for LGBTQ youth. Though the literature has been clear surrounding the risks associated with LGBTQ youth, this scoping review provides a positive outlook on LGBTQ youth’s school experiences and how these systems of social support allow for LGBTQ youth to act as active participants to foster a positive school climate and sense of safety.

**Supplementary Information:**

The online version contains supplementary material available at 10.1007/s44217-022-00016-9.

## Introduction

Lesbian, gay, bisexual, transgender, and queer (LGBTQ) youth spend most of their lives in schools, navigating through the difficult and threatening space [54, 80]. Schools can be a threatening space for LGBTQ youth as they experience increased victimization and a lack of safety [59]. This fact is alarming since students spend most of their time in schools, approximately 175 to 220 days per year with an average of 5 to 8.5 h per school day [81]. Schools, then, can be thought of as youths’ second home, particularly concerning for LGBTQ youth due to the lack of safety in their school environment.

Many studies have indicated that LGBTQ youth experience numerous socioemotional, educational, and health risks at school due to LGBTQ-specific prejudice and victimization. This includes isolation from peers, low social support, low school engagement, low academic success, school dropout, stress, anxiety, depressive symptoms, and suicidal ideation and attempts [41, 47, 60]. However, rather than problematizing youth as at-risk, emerging research is shifting the focus onto the systems that create and carry the risks towards LGBTQ youth, subsequently exploring through a positive lens to begin unpacking LGBTQ needs in schools [54]. Recent research has increasingly focused on positive factors and supports for LGBTQ youth. For example, the presence of a supportive adult in a LGBTQ youth’s lives facilitated a smoother high school experience (i.e., decreased absenteeism, increased academic engagement, [76]). The goal of this study is to systematically explore the positive support systems available for LGBTQ youth, further exploring other potential social support systems, beyond supportive adults, that are present in schools to mitigate the risks for LGBTQ youth and promote positive outcomes. This study will begin by outlining Bronfenbrenner’s [15] Ecological Systems Theory as an approach to understanding social support for LGBTQ youth. A cursory review of the protective factors and stress experiences for LGBTQ youth in schools will be explored followed by the process of a scoping review and thematic analysis. Notably, the review seeks to pivot from a deficit lens of LGBTQ youth considered as at-risk toward systems that promote the positive outcomes of LGBTQ youth. Additionally, the acronym LGBTQ will be used primarily when discussing the LGBTQ+ population. However, when applicable, other acronyms will be used to denote specific subgroups. This can include LGB for studies that explore sexual minority individuals only.

### Understanding social support for LGBTQ youth through Ecological Systems Theory

LGBTQ youth experiences have been increasingly explored in a variety of settings: family, community, and school settings. One approach to organize the LGBTQ youth literature is through a broader, systemic lens. Bronfenbrenner’s [14–16] Ecological Systems Theory can provide the systemic lens needed that allows a way of thinking for the study of interconnections among systems. The model views the individual’s development as a complex system of interactions and relationships across multiple systems surrounding the individual. The systems suggested by Bronfenbrenner [14–16] include: (1) microsystem, (2) mesosystem, (3) exosystem, (4) macrosystem, and (5) chronosystem. Briefly, the microsystem consists of the immediate stakeholders that are directly in contact with the individual (e.g., peers, family). The mesosystem includes the interactions between the individual’s microsystems (e.g., parents speaking with educators). The exosystem consists of stakeholders or environments which do not contain the individual and that indirectly influence the individual via their microsystems (e.g., family’s workplace). The macrosystem consists of the cultural components that influence an individual’s development (e.g., class, ethnicity). The chronosystem consists of normative and/or non-normative environmental changes that occur over the lifespan that can influence an individual’s development (e.g., elementary to high school transition, COVID-19 pandemic). An understanding of the various systems surrounding the individual allows for the exploration of the relationships between the systems (e.g., mesosystems). Previous empirical research on youth and LGBTQ studies have applied the Ecological Systems Theory to understand effective ways LGBTQ youth are accessing the necessary support to thrive in their environment. For example, Watson and others [119] interviewed gay-straight alliance (GSA) advisors addressing various topics including school climate-related issues (e.g., sexual and gender-based victimization) that influenced their ability to be advocates for their students. The authors found that sociocultural factors (e.g., public policies), school-based factors (e.g., administrators), and individual factors (e.g., knowledge of LGBTQ issues) were both barriers and facilitators of their ability to be advocates. From the advisors’ perspective, aligned with Bronfenbrenner’s Ecological Systems Theory, they are required to navigate across systems to effectively support their LGBTQ youth. Results were corroborated from other studies that consider the necessity of school counselors to navigate across ecological systems to support the LGBTQ youth in their schools [7]. Other studies focusing more on the community surrounding LGBTQ youth revealed similar navigations across systemic barriers (or facilitators) in their identity exploration. Katz-Wise and others [57] interviewed TGNB (transgender and non-binary folks) youths’ experience in their gender identity exploration, specifically in family and community settings. Eight themes were developed from this study that aligned with the ecological systems: individual factors (e.g., emotions, coping), family factors (e.g., family support), community factors (e.g., general and LGBTQ community experiences), and societal factors (e.g., external forces). Broadly, the Ecological Systems Theory shifts the research focus to a more relational, developmental systems view, acknowledging the interconnectedness of the systems and its associations to the individual (e.g., [15, 16]).

Taken together, this scoping review attempts to explore existing social support for LGBTQ youth in schools through the Ecological Systems Theory. An ecological systems approach in understanding the existing literature on social support for LGBTQ youth can provide an organizational framework necessary to consolidate the comprehensive literature of social support for LGBTQ youth in schools. As the scoping review attempts an initial exploration and organization of existing social support for LGBTQ youth in schools, a deeper exploration on the relationship between the systems will be explored in a separate review.

### School-based protective systems for LGBTQ youth

American Psychological Association [1] published an informational guide summarizing the various school-based protective systems present for LGBTQ youth. Although not comprehensive, the guide listed several support systems available in schools: (1) educators, (2) school policies, (3) gay-straight alliances, (4) inclusive curriculum, and (5) school climate. Briefly, the guide implicated the importance of educators to help create a safe school climate for LGBTQ youth, the need to create and enforce anti-harassment policies, the creation of gay-straight alliances, and the development of LGBTQ-inclusive curriculum. Each system (educators, policies, GSAs, inclusive curriculum) were found to be critical to an establishment of a LGBTQ-affirming school climate, which in turn was shown to help minimize victimization rates and increase sense of safety for LGBTQ youth [54, 122]. Other research similarly suggests the importance of LGBTQ-affirming school climate as a support system to help minimize victimization rates and increase sense of safety for LGBTQ youth [28].

As literature in this field typically examine systems of social support in isolation (e.g., curriculum, teachers, school policies separately), this scoping review aims to provide a more comprehensive search strategy in consolidating the research on the available social support systems for LGBTQ youth in schools. This scoping review attempts to bring together the literature across multiple systems of social support for LGBTQ youth to develop a systemic definition of social support for LGBTQ youth, identify current research across all systems of social support, identify barriers and difficulties experienced by LGBTQ youth in schools, and identify areas for future research in understanding the social support systems for LGBTQ youth.

### Schools as a key site of stress for LGBTQ youth

Results from the 2015 Youth Risk Behavior Survey (YRBS) indicate that over 60% of LGB youth experienced prolonged feelings of hopelessness compared to only 25% of heterosexual youth. In a national survey of LGBTQ youth [59], 67% heard homophobic comments in schools, 58% perceived a lack of safety as a result of their sexual orientation identity, and 43% perceived a lack of safety as a result of their gender identity and expression. Although there was a high percentage of LGBTQ-specific concerns, only 12% of LGBTQ youth reported teacher intervention. In Gay Lesbian Straight Education Network (GLSEN)’s national survey [59], 92.6% of LGBTQ youth mentioned health concerns (e.g., depression, anxiety) as the main reason for not graduating high school, followed by academic (e.g., poor grades, absences), and safety concerns (e.g., hostile school climate, harassment, unsupportive peers and staff). Therefore, a further detailed systematic breakdown of existing social support systems in educational settings is required to better understand what can be done to offset negative experiences and risks. This analysis will also clarify the barriers schools face in providing support and inform future inquiry for schools to move towards improved support for LGBTQ youth.

Present data highlights that LGBTQ youth are at a heightened risk for numerous health and educational concerns. Such concerns can be attributed to a lack of connection with their teachers and school staff [58], a lack of acceptance from their family members [57, 74] and peers [122], a lack of school curricula and policies that value LGBTQ diversity [105], and the existence of overall hostile and exclusionary school climates [59]. The level of warmth and positivity in a school environment can positively impact LGBTQ students’ experiences and their subsequent health and educational outcomes. For example, teacher-student relationships are positively associated with increased academic engagement, performance, and social-emotional wellbeing for LGBTQ youth [66]. This review seeks to pull together literature on how LGBTQ youth are supported in schools and examine the ways that different types of social support can affect outcomes to provide an organized framework to effectively support LGBTQ youth.

### Research question and aims of the current study

While efforts have been made to support LGBTQ youth in schools, literature is diffuse and show mixed results [54, 109]. Subsequently, a systematic surveying of the literature on all existing support systems that provide the necessary social support for LGBTQ youth is necessary. Social support includes numerous school professionals and community members such as school psychologists, educators, counsellors, and principals to act as critical individuals holding the power to support and advocate for LGBTQ youth. The scoping review aims to synthesize current research on social support for LGBTQ youth in schools. Recurring literature on social supports for LGBTQ youth include gay-straight alliances (GSAs), school policies, curriculum, and parent and peer support [54]. The review seeks to direct future research by providing clarity and illuminating gaps in literature to foster more nuanced research and interventions that ameliorate significant health and educational disparities for LGBTQ youth. As research is robust, indicating the disproportionate stress that LGBTQ youth experience [122], this review is imperative to systematically explore the systems of social support for LGBTQ youth.

This study seeks to respond to the following question:

How does social support in elementary and secondary education relate to outcomes for LGBTQ youth?

with the following objectives:

1. Define what it means to have social support in schools,
2. Identify and describe the current research on outcomes for LGBTQ youth given the implementation of these social support systems,
3. Identify barriers and difficulties to support LGBTQ youth in an educational setting, and
4. Identify areas for future research for LGBTQ youth and social support in schools.

## Method

### Search strategy

This study follows the methodologically rigorous scoping review approach designed by Arksey and O’Malley [5] and conducted a systemic search across the disciplines of education and psychology. Though the keywords and categories used to conduct the systemic search was all-encompassing and should capture relevant stakeholders in schools, specific domains of studies outside of the field of education and psychology was not explicitly considered due to the interdisciplinary nature of education. Therefore, the search strategy may not have a wide reach for paraprofessionals that work with LGBTQ youth in schools (e.g., social workers). However, based on the broad nature of the keywords, paraprofessionals working in schools to support LGBTQ youth should be included. A scoping review was chosen to allow for the inclusion of multiple study designs and to allow for post-hoc analysis of inclusion and exclusion criteria [86]. In particular, as a systematic review approach required study appraisals, a scoping review was more appropriate due to the inclusion criteria of both empirical and non-empirical studies.

### Eligibility criteria

A set of inclusion and exclusion criteria were established a priori to provide guidance for the systematic search strategy. Inclusion criteria included: (a) empirical articles that were published in peer-reviewed journals between 2007 and 2021; (b) non-empirical literature including books, book chapters, case reports, reviews between 2007 and 2021; (c) written in the English language; (d) LGBTQ individuals; (e) school environment-specific (ranging from elementary through high school, including technical schools); (f) all geographical locations; and (g) social support outcomes for students. Exclusion criteria included: (a) non-LGBTQ specific; (b) unrelated to school environment; (c) social support outcomes not for students (i.e., teachers, parents).

### Information sources

The search used the following databases: PsycINFO, ERIC, Genderwatch, ProQuest Dissertation and Thesis, Web of Science, Cochrane Database of Systematic Reviews, and Campbell Systematic Review. A description of keywords can be seen in Supplementary Table 1 and a visual for the search and data collection process in Supplementary Figure 1.

### Search

A social science librarian was consulted to ensure the scoping review was conducted in a systematic procedure. The five databases were cross-checked with an expert in the field of LGBTQ studies to ensure a comprehensive collection of databases. After databases were confirmed, key concepts were brainstormed and cross-checked with the second and third author, the expert in the field of LGBTQ research (April 2017), and the librarian (May 2017). Keywords were broken down into three sections. The first column consists of LGBTQ terms (e.g., homosexuality, bisexuality, gender identity, transgender or [attitudes towards]). The second column consists of school terms (e.g., high school students). The last column consists of social support terms (e.g., peers). Refer to Supplementary Table 1 for a full list of search terms. All keywords in each column were combined. After a collaborative process between the authors, librarians, and expert, all keywords and related terms were included in each database.

### Data collection process

Data were collected during June 2017 and revised in February 2021 to ensure consistency between the searches. Throughout the collection process, the authors engaged in an iterative process to discuss obstacles that arose during the screening phase. As depicted in the flow chart (Supplementary Figure 1), the initial data collection yielded 565 articles (*n*_2017_ = 364; *n*_2021_ = 199). After deduplications were removed, 533 articles remained (*n*_2017_ = 335; *n*_2021_ = 198).

### Phase one: title and abstract screening (2017)

Phase one consisted of an initial screening of the relevant literature. During this phase, the first, second, and third authors conducted independent title and abstract screening of the 335 articles, resulting in an interrater agreement of 71.94%. Any disagreements across the authors were discussed until a consensus was reached based on the inclusion/exclusion criteria. Following the screening, 128 articles remained.

### Phase two: full text screening

An independent screening by the first, second, and third authors of the 128 articles identified in phase one resulted in 54 articles being retained in the review. Interrater agreement was 80.47%. Consensus was achieved through iterative discussion among the authors to determine the final literature count.

### Phase three: data extraction

Once the final sample of studies were selected, a table was created to depict important information from each study: (1) study characteristics (e.g., study design, school setting, research question), (2) group demographics (e.g., LGBTQ acronym, sample size, grade level, age range), (3) social support factors, and (4) key findings.

The resulting 54 articles from the full-text screening were broken into three blocks of 18. Each author independently read two of the three blocks of articles and extracted relevant data (such that the first author independently read blocks A and B; the second author independently read blocks A and C; and the third author independently read blocks B and C). After independent data extraction, the two reviewers for each corresponding block resolved any differences.

### Phase four: Revised data collection (2021)

A revised data collection was addended since the 2007 through 2017 phase. Another round of data collection, abstract, and full-text screening was conducted from 2017 through 2021. The update in data collection was done in 2021 to account for the many changes happening in society related to LGBTQ populations (e.g., anti-LGBTQ bills; [95]) as the manuscript was in the process of writing and revisions, along with the delays as a result of the COVID-19 pandemic. An additional 198 articles were collected for initial screening (totalling 533 articles, see Supplementary Figure 1). Following the same procedures of phase one title and abstract screening, independent screening was conducted by the first and fourth author, resulting in 56 articles retained with an inter-rater reliability of 84.34% (*N*_2017+2021_ = 184). Replicating phase two, the first and fourth author conducted independent full-text screening on the 56 articles, resulting in 40 articles with an inter-rater reliability (IRR) of 75.00% (*N*_2017+2021_ = 94). Following phase three, the resulting 40 articles from the full-text screening were broken into two blocks of 20. Each author independently read one block of articles and extracted relevant data. After independent data extraction, the two authors checked and resolved any differences in the other block.

### Synthesis of results

After data abstraction, quantitative data was collected on the following categories (see Supplementary Table 2): (1) research design, (2) participant sample size range, (3) LGBTQ acronym, (4) school setting, (5) number of schools, (6) number of students, and (7) the types of social support. Initial IRR of 94 articles was 76.60% and discrepancies were discussed and resolved through an iterative process between the first through fourth authors.

Subsequent thematic analysis [3, 13, 25, 36, 117] was conducted. This method of analysis is justified as a descriptive, qualitative method to identify common themes found in the key findings of the 94 articles. Initial IRR was 78.72%, above the acceptable level of reproducibility, and discrepancies were discussed and resolved among the first four authors.

Data analysis involved both quantitative (e.g., frequency analysis) and qualitative (e.g., thematic analysis) methods, resulting in a multi-layered synthesis process that allowed for the identification of existing gaps in the literature and revealed potential topics for conducting future systematic or novel reviews.

## Results

### Study characteristics

Refer to Supplementary Table 2 for a tabulation of characteristics across the 94 articles.

### Research design

Out of the 94 articles, there were 48 (51.06%) quantitative studies, 43 (45.74%) qualitative studies, and three (3.19%) mixed-methods studies.

### LGBTQ acronym

As each article used several LGBTQ acronyms interchangeably, there are a total of 102 acronym frequencies across 94 articles. Acronyms include LGBTQ/GLBTQ (*n* = 40; 39.22%), LGBT/GLBT (*n* = 15; 14.71%), sexual minority/SMY (*n* = 10; 9.80%), LGBQ (*n* = 7; 6.86%), LGB/GLB (*n* = 6; 5.88%), transgender/trans* (*n* = 4; 3.92%), SSA (*n* = 3, 294%), GSM/GSD (*n* = 3; 2.94%), LGBTQQ (*n* = 2; 1.96%), gender-variant (*n* = 2; 1.96%), GM (*n* = 2; 1.96%), LGBTQ2S (*n* = 2; 1.96%), LGBTQ+ (*n* = 2; 1.96%), queer (*n* = 1, 0.98%), MSMY (*n* = 1; 0.98%), bisexual/pansexual (*n* = 1; 0.98%), TGD (*n* = 1; 0.98%).

### Participant sample range

Across 94 articles, 42 studies provided specific age or grade ranges of the participants. Participants ranged from students in grades nine through 13 (*n* = 20; 21.28%), grades seven through 12 (*n* = 10; 10.64%), grades eight through 12 (*n* = 6; 6.38%), grades 10 through 12 (*n* = 4; 4.26%), and grades six through 12 (*n* = 2; 2.13%). 40 studies did not provide specific age or grade range of students and only included the educational institution broadly: high school (*n* = 16; 17.02%), middle and high school (*n* = 7; 7.45%), high school and college (*n* = 5; 5.32%), middle school (*n* = 4; 4.26%), elementary school (*n* = 4; 4.26%), college (*n* = 3; 3.19%), elementary and high school (*n* = 1; 1.06%). The remaining 12 studies included adult staff or parent participants (*n* = 5; 5.32%) or did not specify (*n* = 7; 7.45%).

### School setting

As each study recruited school settings that were different in type (i.e., catholic, private, democratic) and in developmental age (i.e., elementary, middle, high school), there was a total of 108 counts of school settings across the 94 articles. School settings included high school (*n* = 46; 42.59%), middle and high school (*n* = 28; 25.93%), private schools (*n* = 5; 4.63%), elementary through high school (*n* = 4; 3.70%), elementary school (*n* = 4; 3.70%), catholic schools (*n* = 4; 3.70%), middle school (*n* = 3; 2.78%), college (*n* = 3; 2.78%), alternative schools (*n* = 2; 1.85%), community center (*n* = 1; 0.93%), democratic school (*n* = 1; 0.93%), and independent school (*n* = 1; 0.93%). Six studies (5.56%) did not specify the type of school setting.

### Types of social support

Each study reported more than one type of social support related to LGBTQ students, resulting in a total of 188 counts of social support types. Social support was organized into four categories: school support (*n* = 139; 73.94%), peer support (*n* = 24; 12.77%), parental support (*n* = 16; 8.51%), and community support (*n* = 9; 4.79%). School support was further broken to include gay-straight alliances (*n* = 42; 22.34%), supportive non-teaching staff (*n* = 34; 18.09%), supportive teachers (*n* = 24; 12.77%), positive school climate (*n* = 12; 6.38%), programs and policies (*n* = 11; 5.85%), school-wide approaches (*n* = 9; 4.79%), and curriculum (*n* = 7; 3.72%).

### Synthesis of results

Based on Bronfenbrenner’s Ecological Systems Theory, the constructed themes that was developed across the 94 articles were organized into support systems that directly impact LGBTQ youth outcomes (see Supplementary Table 3). As geographical information was not extracted, findings are generalized and may not accurately represent specific geographically contextualized policies and environments.

### The role of family (caregiver) systems and social support

Three distinct themes were constructed from the literature: (1) high actual or perceived family/caregiver support buffered many negative socioemotional or educational outcomes (*n*_articles_ = 12), (2) family/caregiver support was not consistently adequate to buffer the negative emotional, behavioral, and educational outcomes (*n*_articles_ = 3), and sex differences within family experiences highlighted complexities of family/caregiver support (*n*_articles_ = 3).

#### High caregiver support buffering negative outcomes

When family (or caregiver) support was low, LGBTQ youths’ level of emotional and behavioural distress was high [4, 8, 18, 24, 40, 55, 85, 90, 124]. A lack of social support in the family system (e.g., family harassment, low caregiver support, low communication and closeness) was positively associated with adverse social (e.g., disengaging from peers, running away from home [40, 55, 85, 124]), emotional (e.g., depression, psychological distress, substance abuse, suicidal ideation [4, 8, 18, 55, 85]), and educational outcomes (e.g., school dropout [8]), for LGBTQ youth. However, studies have shown that family acceptance was a type of social support that fostered LGBTQ youths’ critical thinking and advocacy for safe spaces in schools to support marginalized students [40, 124]. Family support was particularly associated with better school performance for LGBTQ racialized youth. For both White and racialized LGBTQ youth, perceptions of being close with parents and direct involvement with parents in activities moderated experiences of victimization at school, and reduced substance use and suicidality, educational risks, and increased school belonging [18, 85, 90]. Moreover, LGBTQ-affirming resources aimed at developing family support (e.g., parent advocacy, allyship, communication, trust) fostered LGBTQ youth academic well-being, physical and emotional safety, and ability to be authentic in classrooms [23, 38, 82].

#### Caregiver support inconsistent in buffering negative outcomes

Studies showed that family (or caregiver) support did not consistently buffer the negative outcomes that happens at school [17, 90]. Though family support may be protective against victimization and self-harm among youth, effects were less robust for gender minority youth [96].

#### Sex differences within family experiences

Three unique studies found differences present for (1) boys and girls and (2) mothers and fathers. Pearson and Wilkinson [85] found that only sexual minority girls were less distressed when they reported a sense of strong family relationships. However, there was no association found between caregiver support and peer victimization for sexual minority girls [55]. Bos and others [11] found less distress among all LGBTQ youth who established a strong relationship with their fathers (e.g., more disclosure and communication but not their mothers). A strong relationship with fathers resulted in increased positive social (e.g., more peer acceptance), emotional (e.g., increased self-esteem, decreased depression), and educational outcomes (e.g., increased school belonging, [11]).

### Supporting LGBTQ youth through the curricular education system

Four distinct themes were constructed from the literature: (1) LGBTQ-inclusive curriculum was most often taught in social sciences, humanities, and health classes, fostering authenticity with students and creating an inclusive classroom (*n*_articles_ = 6), (2) LGBTQ-inclusive curriculum led to decreased victimization and negative socioemotional outcomes and increased sense of safety (*n*_articles_ = 5), (3) a hidden, heteronormative curriculum exists behind the official academic curriculum that impedes LGBTQ youth support and engagement (*n*_articles_ = 4), and (4) a need for teachers to feel supported to teach LGBTQ-inclusive curriculum effectively (*n*_articles_ = 4).

#### LGBTQ-inclusive curriculum fostering authenticity with students and creating an inclusive classroom

LGBTQ-inclusive curriculum appeared to be taught only in specific classes, specifically in social sciences, humanities, and health classes [10, 103–105, 124]. Making connections with LGBTQ-inclusive material allowed students to make authentic connections between their lives and the class content [73] which contributed to an increased psychological wellbeing and disrupted homophobia and other forms of oppression [10, 103, 118, 124]. Teachers who incorporated LGBTQ material into their curriculum allowed youth to identify teachers as possible safe adults to discuss sensitive concerns (e.g., LGBTQ-related concerns, coming out). Teachers also agreed on the importance of weaving social justice topics in the curriculum to model critical literacy and to create an inclusive curriculum, benefitting all students [84].

#### LGBTQ-inclusive curriculum decreased negative outcomes and increased sense of safety

LGBTQ-inclusive curricula had supportive elements at the individual and school level (i.e., increased feelings of safety at school, decreased feelings of isolation and depression, and more awareness of victimization at school; [70, 105]). Incorporating LGBTQ-inclusive curriculum and having access to LGBTQ-related information in schools was positively associated with perceptions of a safer school environment and negatively associated with perceptions of victimization [105, 110]. Therefore, developing a curriculum that centers LGBTQ issues can disrupt homophobia, injustice, and other forms of oppression, which can provide safety and acceptance, and validate LGBTQ youths’ experiences at school [102, 118].

#### Hidden, heteronormative curriculum impedes LGBTQ youth support and engagement

This theme expands on the hidden, heteronormative curriculum that exists behind the official academic curriculum. Castro and Sujak [19] mentioned the need for LGBTQ-inclusive curriculum to expand outside of academics, such as the social (e.g., relationships and communication) and campus curriculum (e.g., inclusive group space). LGBTQ-inclusive curriculum is most effective when it can be generalized beyond formal learning spaces. Gay-straight alliances (GSAs), a supportive network outside of the classroom, is one space that can supplement LGBTQ-inclusive curriculum outside formal education. Informal spaces of LGBTQ-inclusive curriculum can foster student engagement and provide further opportunities for students to engage in social advocacy and promote a positive school climate [64, 73, 123].

#### Teachers need to feel supported to teach effective LGBTQ-inclusive curriculum

Though LGBTQ-inclusive curricula can be a pillar of social support for LGBTQ youth, teachers often miss teachable moments conducive to inclusive curriculum [70, 103]. Teachers mentioned difficulty fostering an inclusive curriculum due to rigid curriculum, high stakes testing, and parental resistance [84], requiring the administration to provide the support needed for teachers to change the curriculum [69]. Note that the barriers may be contextual as high-stakes testing does not occur in all school contexts and curricula may be externally constructed in relation to the geographical context of the school environment.

### Gay-straight alliances (GSAs) and other school programs

Six distinct themes were constructed from the literature: (1) gay-straight alliances (GSAs) fostered a space for empowerment and change, creating a safe space and climate for LGBTQ youth (*n*_articles_ = 24), (2) GSAs created opportunities for connection for LGBTQ youth in their community (*n*_articles_ = 13), (3) GSAs allowed for engagement and youth involvement in schools (*n*_articles_ = 11), (4) GSAs had varying functions (*n*_articles_ = 7), (5) GSAs encountered challenges in delivering positive outcomes (*n*_articles_ = 15), and (6) school-based interventions (non-GSAs) were effective in supporting LGBTQ youth (*n*_articles_ = 6). Note that most of the articles referred to GSAs as gay-straight alliances. One article referred to them as gender-sexuality alliances.

#### GSAs foster a space for empowerment and change, creating a safe space and climate for LGBTQ youth

GSAs help students provide a space to act together to create cultural and institutional change [31, 98, 123] and can be transformative for school culture. These spaces provide a positive and safe physical and intellectual space where students can engage in knowledge transfer and discuss LGBTQ issues otherwise silenced in the larger school community [31, 40, 63, 64, 69, 71, 72, 77, 78, 98, 106, 107, 123]. GSAs give LGBTQ youth a safe place to go where they can be accepted [69]. GSAs can be a space where mental health promotion programs can be incorporated to provide students with coping skills and resources [44]. The presence and membership in GSAs were positively associated with school belongingness, school engagement, school safety, academic success, wellbeing, and negatively associated with substance use, psychological distress, and victimization incidents [6, 45, 46, 53, 67, 93, 111, 112]. Entering GSA classrooms offered visibility, positive symbols of acceptance, respect, and affirmation, providing LGBTQ youth with a sense of safety [6, 87].

#### GSAs create opportunities for connection for LGBTQ students in their community

GSAs provide accountability, support, community, increased academic success, and decreased feelings of isolation by connecting youth with other LGBTQ community members, events, and resources. Subsequently, the connections lead to increased validation and normalization of identity, sense of hope, acceptance, greater self-esteem, greater appreciation for self and other peers, adaptive social relationship skills, and a sense of safety and empowerment for LGBTQ youth [31, 40, 46, 69, 71–73, 75, 98, 106, 111]. GSAs allowed for connections to community organizations, providing a gateway to the wider LGBTQ community, supportive adults, community resources, fostering activism opportunities and increasing LGBTQ visibility [6, 87].

#### GSAs allow for engagement and youth involvement in schools

Participation in GSAs were positively associated with perceptions of a safer space for LGBTQ youth to engage in self-expression and identity validation [62]. Their involvement in GSA-related activities and events increased their self-efficacy [20], academic success, school engagement, school belongingness [43, 111, 112], sense of hope, and advocacy and awareness-raising efforts [88, 91]. Engaging with GSAs enabled students to form their own identities grounded in empowerment rather than as victims [31, 98]. LGBTQ youth, teachers, and school administrators have reported that having and engaging in their GSA gave students space for emotional safety [71, 72].

#### GSAs vary in their function (e.g., advocacy, educational, socialization)

GSAs had distinct purposes in assisting different aspects of LGBTQ youth: (1) advocacy, education, and social support; (2) literature to reflect on the lives and experiences of LGBTQ youth; and (3) developing skillsets to assist students in fostering inclusion and acceptance [114]. Advisors believed the primary role of GSAs is to bring awareness and act in schools, whereas students believed the purpose was to foster a sense of community and belongingness [63, 71, 94]. The varying functions of GSAs depended on the internal provisions of support, from visibility raising to collective social change [71, 92, 123]. Students who were more involved in accessing information and advocacy efforts discussed more health-related topics, prepared more awareness-raising campaigns, and had increased school engagement [89]. On the other hand, GSAs with a stronger focus on socialization efforts focused less on mental health discussions [89].

#### GSAs encounter challenges in delivering positive outcomes

Although GSAs were found to be effective in supporting LGBTQ youth in schools, only 19.1% of youth reported an existence of a GSA in their high school [8]. Program implementations within GSAs also encountered common problems. Problems included a lack of staff training and safe staff, a lack of student understanding towards LGBTQ issues, a lack of sensitivity towards LGBTQ topics, and challenges in facilitating a discussion on sexual or gender-related topics [50, 69]. GSAs struggled to subvert the heteronormative school climate in schools where the greater community was unsafe, particularly in rural environments [28, 63, 71]. For example, high schools had concerns and restricted policies on GSA student behaviours, limiting activities allowed by students [31, 35]. In communities that were indifferent or hostile towards LGBTQ populations, GSA advisors were required to negotiate with school administrators to provide LGBTQ youth a safe space in schools [6]. In schools with high levels of victimization, the benefits of GSA-related social justice involvement and presence dissipated [111, 112]. In some schools, the presence or participation in GSA activities did not predict student school engagement and was not associated with mental health outcomes or sense of safety [21, 28, 93, 99, 100]. Rather, the presence of a GSA led to emotional vulnerabilities to the wider school community [6]. As such, the impact of GSAs on LGBTQ youth safety and school climate may vary widely across schools and geographic context.

#### School-based interventions (non-GSAs) were effective to support LGBTQ youth

There is a need to employ a pragmatic approach and focus on student safety to gain administrative support to conduct interventions [65, 69, 94]. Classroom intervention focused on accepting individual differences through open discussion and participation of emotional and sensitive issues were effective in framing uniqueness as a strength and fostered change towards an accepting classroom climate [70, 94]. Youth-led theater and dialogue-based interventions were effective to address heterosexism and genderism in schools, with increased reports of willingness and intention to advocate for social justice and equality for LGBTQ people [121]. Hall and others [42] showed how a student-led community art gallery was effective to create a space for discussion on gender issues and act towards supporting LGBTQ youth.

### The role of peer systems in supporting LGBTQ youth

Two distinct themes were constructed from the literature: (1) peer support and acceptance were related to lower levels of emotional and behavioural distress and fostered positive outcomes (*n*_articles_ = 13), and (2) inconsistencies in the effectiveness of peer support for diverse LGBTQ youth (*n*_articles_ = 4).

#### Fostering peer support and acceptance relates to lower levels of emotional distress and fosters positive social and educational outcomes

LGBTQ youth who had higher levels of peer acceptance and lower levels of strained peer relationships experienced lower levels of depression and suicidal behaviour, higher levels of self-esteem, increased academic success [11, 51, 56], particularly for youth from rejecting families [23]. On the other hand, lower peer acceptance or connection predicted higher levels of depressive symptoms and lower levels of self-esteem and belongingness to the school [11]. Uniquely, peer acceptance from straight allies played an important role to address anti-gay stereotypes [64]. Engaging in peer education and interventions led to increased levels of safety for LGBTQ youth [28, 33, 102]. Older youth were found to have less homophobic attitudes and were more willing to remain friends with GL youth [110]. Schools where GLB youth had opportunities to socialize reported increased belonging in their school and in their larger community [79]. Being out (i.e., disclosure of gender or sexuality) to more peers at school was generally associated with higher grades and less school harassment [120]. Similarly, seeing peers who were out was positively associated with a sense of safety in schools [83]. Having thick friendships were shown to help encourage LGBTQ youth to question their sexuality [37]. The culmination of research on peer support reiterates the importance of peer support in schools for an increasingly safe and positive school environment.

#### Inconsistencies in the effectiveness of peer support for diverse LGBTQ youth

Though peer support was effective in fostering positive socioemotional outcomes and minimizing emotional distress, inconsistencies were found within the LGBTQ community. Sub-group identities had different conclusions regarding the effectiveness of peer support. Craig and Smith [24] found that racialized LGBTQ youth did not have a relationship between peer support and educational outcomes. Studies show that having supportive peers to discuss problems increased the risks of suicidal ideation and attempts for LGBTQ youth, particularly for LGBQ youth who have had been victimized and gender minority youth [17, 96]. Generally, social support did not buffer effects of victimization on self-esteem for LGBTQ students [108], questioning the nuances in the efficacy of peer support as a social support system.

### School professionals and teachers as a system of support for LGBTQ youth

Four distinct themes were constructed from the literature: (1) high level of within-school adult support resulted in positive benefits (*n*_articles_ = 19), (2) high level of within-school adult support reduced negative outcomes (*n*_articles_ = 10), (3) teachers and school staff were ineffective and inconsistent in supporting LGBTQ students (*n*_articles_ = 7), and (4) school staff perceived external support as key to ensure coordination of inclusivity for LGBTQ students (*n*_articles_ = 4).

#### High level of within-school adult support results in positive benefits

LGBTQ youth perceived more support in schools when they perceived that their school staff, administrators, and teachers showed more than verbal support (i.e., lip service). LGBTQ youth mentioned the need to observe school staff acting and having a presence explicitly taking a stance against bigotry, emphasizing the importance of behavioural management to establish a safe classroom space [10, 69–71, 84]. LGBTQ youth who had natural mentors (e.g., teachers, staff members, school administrators) were three times as likely to graduate from high school, had increased intentions to seek help for suicidal thoughts [21], and had positively impacted their engagement and connectedness to their school [23] compared to youth who did not have such mentors [30]. When the number of “safe adults” increased at school, LGBTQ youth would become more engaged with their school and community through opportunities and access to resources from supportive staff members [69, 72, 99, 100]. Supportive teachers had the power to foster a safe classroom climate and environment, set clear expectations, open inclusive dialogue with students, implement LGBTQ-inclusive school and classroom procedures that positively impacted LGBTQ youths’ safety and acceptance in schools [28, 102], educational achievement [34], and wellbeing [116]. Teachers having power to foster a safe classroom climate was similarly voiced by TGNC youth, subsequently supporting their transition [38]. Likewise, teachers and school staff understood the importance of developing skill sets (e.g., use of inclusive language) to foster an inclusive and supportive classroom environment for LGBTQ youth [113, 114]. Therefore, supportive school staff are key stakeholders to foster a safer classroom environment and to create opportunities to foster awareness of LGBTQ issues in their school environment (i.e., creating a community art gallery, [42]).

#### High level of within-school adult support reduces negative outcomes

LGBTQ youth perceptions of greater adult support (i.e., principals, social work professionals, teachers, school administrators) at school was linked to lower levels of victimization, school avoidance, substance use, suicidal behaviour, and other mental health risks (depressive symptoms; [21, 23, 26, 51, 101]). The identification of an adult ally predicted a decrease in fear-based truancy [72, 99, 100]. Principals agreed that there is a need to increase efforts to reduce discrimination towards LGBTQ youth by setting a safe and positive climate in schools [12]. An avenue that was effective in creating an inclusive and affirmative environment and reduce health risks among LGBTQ youth are school-based health centers [125].

#### Teaching and non-teaching school staff were ineffective in supporting LGBTQ students

Though there are benefits in having a supportive school staff, there was a lack of communication between LGBTQ youth and school staff. 80.9% of LGBTQ youth reported never talking to a teacher about LGBTQ topics, 70.8% of youth never talked to a school health counselor, and 86.5% of youth never talked to a school administrator about LGBTQ issues in school [8]. The lack of action or silence teachers and school administrators take towards LGBTQ topics or incidents is a reason for the lack of communication. Students reported that teachers are inconsistent in their intervention against victimization incidents, often focused on stopping the harassment and providing reasoning for why such incidents can cause harm [48]. There was a common perception of school administration silence surrounding LGBTQ topic as normative in school environments [71]. Teachers reported feeling unprepared to support LGBTQ youth and required more information, for example, through collaboration with GSAs to improve pedagogy [70, 72]. Coulter and others [22] found that within-school adult support was ineffective in protecting LGBTQ youth against suicidality compared to outside-school adult support. Therefore, teachers and school staff need to increase their responsibility to support LGBTQ youth [38].

#### School staff perceived external support as necessary to foster staff support for LGBTQ students

School staff mentioned the importance of having a coordinator external to the school to provide support for curricular efforts and activities to students and staff, and adapting to school needs, reducing harassment for LGBTQ youth [50, 69, 70]. Schools with an external source of support (i.e., external staff) showed significant improvements towards supporting LGBTQ youth, as reported by student observations [50]. Sexuality education workshops were another form of external support that led to significant positive effects on teachers’ beliefs and behaviours to support their LGBTQ youth [61].

### The role of school policies and safer school spaces for LGBTQ youth

Three distinct themes were constructed from the literature: (1) socio-political values of the wider community beyond the school impacted school policies and staff attitudes (*n*_articles_ = 7), (2) implementation of inclusive and anti-discriminatory policies were effective in fostering a safer school space for LGBTQ students (*n*_articles_ = 5), and (3) school policy and community support showed challenges in fostering positive outcomes (*n*_articles_ = 4).

#### Socio-political values of wider community impacting school policies and attitudes

Policies from the broader context can provide the support needed for schools to have inclusive school policies. Supportive government and school board policies allowed for organizations (i.e., GSAs) to be accepted, subsequently fostering community connection and support for LGBTQ youth [69, 106]. However, schools located in communities with more non-progressive attitudes and beliefs about LGBTQ individuals due to political or religious conservatism generated hesitation to support LGBTQ students by school administrators [65, 71, 124]. Hesitations to support LGBTQ youth include the ban of GSA creation, sending a message regarding LGBTQ invisibility in school environments [65]. School staff were cautious and focused on minimizing external resistance and pressure from the larger community. As a result, this led to restrictions in GSA activities and spaces [65, 71]. Snapp and others [104] found that school policies were inequitably enforced as LGBTQ youth were punished for public displays of affection and violation of dress code compared to heterosexual peers, indicative of a lack of inclusive school policies.

Reframing the support for LGBTQ youth as systematic inclusion to meet the needs of all students may be a method to circumvent the restrictions and pressures from the larger community environment. Reframing support for LGBTQ youth to general support for all students can reduce the hesitance school staff have to support LGBTQ youth [65, 71, 106]. Most notably, a school-wide approach and communal investment is required to change and move towards inclusive school policies, promoting the social, psychological, and physical safety for all students [33].

#### The implementation of inclusive and anti-discriminatory policies to foster safe school spaces

Schools with higher reported implementation of inclusive and anti-discriminatory policies had lower levels of discrimination against LGBTQ youth [12], fostering a safer school space. Effective bills such as Bill 13 (i.e., Accepting Schools Act, Ontario, Canada) allowed LGBTQ youth to create a space to transform their lives and offer opportunities of activism [52]. Inclusive policies allowed for inclusive events (i.e., Pride Prom, Day of Silence) that provided a safer environment for LGBTQ youth [107]. Therefore, inclusive policies are important to set up a safe environment for students and challenge the hetero/cisnormative dynamic present in policy documents and classroom environment [113]. Introducing inclusive policies require collaboration across professionals to support legislation that acknowledges LGBTQ issues in schools [61].

#### Inconsistencies in fostering positive outcomes from inclusive school policies and wider community support

Bullying policies did not consistently predict LGBTQ safety and victimization [12, 28]. Rather, higher proportions of students who reported inclusive school policies predicted lower perceptions of safety based on gender nonconformity [110]. Lastly, community support was not related to decreased rates of harm for LGBTQ youth [96].

### The role of a positive school climate on LGBTQ youth outcomes in school

Three distinct themes were constructed from the literature: (1) a positive school climate reduced negative emotional-behavioural outcomes (*n*_articles_ = 4), (2) a positive school climate fostered positive psychosocial and educational outcomes (*n*_articles_ = 11), and (3) a whole school effort is required to foster a positive school climate (*n*_articles_ = 10).

#### Positive school climate reducing negative emotional-behavioural outcomes

For both LGBTQ and heterosexual youth, a positive school climate, strong school connectedness, and involvement in school-based activities predicted fewer physical victimization, fewer depressive symptoms, less suicidal ideation and attempts, substance use, and truancy [9, 21, 29, 32]. Similarly, teachers reported perceiving fewer depressive symptoms among their male sexual minority youth in positive and supportive school environments [29].

#### Positive school climate fostering positive psychosocial and educational outcomes

A positive and safe school climate (e.g., GSA activities; LGBTQ-affirming school-wide campaigns) can promote tolerance, respect and inclusion for LGBTQ youth [69, 72, 121]. LGBTQ youth who were in less heteronormative schools, had inclusive classroom environments, and LGBTQ affirming school climates allowed them to be more inclusive, have increased opportunities to understand diversity and differences [102], fostered increased psychological wellbeing [116], and had more positive perceptions of safety in their schools [28]. A positive school climate has also benefitted teachers by helping them feel comfortable to advocate for their LGBTQ youth [70, 72]. Students, parents, and school staff mentioned the importance of having a safe space as a deciding factor to attend school for students to be recognized, accepted, and to participate in their school [49]. Subsequently, those who were more involved in school activities and had stronger school connectedness felt safer in schools and had increased achievement [32, 34, 100].

#### Whole school effort is required to foster a positive school climate

Creating and maintaining a positive and safe school climate for LGBTQ youth can foster positive outcomes for all students. This effort requires constant vigilance from all relevant stakeholders: students, teachers, administration, and community members [94]. Effective interventions (LGBTQ-inclusive curriculum, GSAs, supportive school staff, staff development and training, awareness events, appropriate mental health services, inclusive policies, inclusive language, school-home-community connections, and community partners) are all necessary to foster a positive school climate. This, in turn, provides support for LGBTQ youth and fosters wellbeing, and educational and social success [39, 50, 56, 83, 94, 121, 123, 124]. A concerted effort provides LGBTQ youth with access to resources and create more opportunities to carry out programs and training that can maximize the potential for LGBTQ youth to feel supported in their wellbeing and safety. Additionally, a whole-school approach can support teachers and school administrators by providing them with more resources and external support, all instrumental to attain a whole-school system that is positive and inclusive [69]. GSAs may be an avenue whereby students can act to address anti-LGBTQ bias, to provide education, and address the silences on LGBTQ issues through whole school efforts [69, 121, 123]. Most importantly, having a supportive principal can facilitate a positive whole-school approach to promote LGBTQ inclusivity in schools [70].

## Discussion

### A systemic definition of social support for LGBTQ youth

The first objective of this review is to define what social support in schools mean for LGBTQ youth. Prior to understanding how social support in elementary and high school education relate to outcomes for LGBTQ youth, the scope of social support needs to be defined to create a systemic framework that can map how different social support systems are associated with LGBTQ youth outcomes in school.

Organized through the Ecological Systems Theory, social support can be defined as support that is provided across various systems related to LGBTQ youth. This scoping review brought forth how social support in schools for LGBTQ youth can span across systems: (1) family, (2) curriculum, (3) GSAs (and other school programs), (4) peers, (5) school administrators and teachers, (6) school policies, and (7) school climate.

The seven systems that were constructed from the review indicated that they impact LGBTQ youth and their experiences in school. The parental system was constructed from the review as a form of social support that is associated with LGBTQ youth outcomes in schools. Parents or caregivers who supported their LGBTQ youth through advocacy, open communication, trust, closeness, and acceptance minimized many negative educational outcomes (i.e., depressive symptoms, substance use, victimization) and promoted wellbeing, academic success, physical and emotional safety among other outcomes. The curricular system was constructed to show how influential LGBTQ-inclusive curriculum can be for LGBTQ youth. LGBTQ-inclusive curriculum provided LGBTQ youth the opportunity to explore their LGBTQ identity, make authentic connections, challenge oppression, and acquire knowledge inclusive of LGBTQ people and issues. When a LGBTQ-inclusive curriculum is introduced in classrooms, LGBTQ youth reported feeling safer, more accepted in their classroom, and lower victimization incidents. GSAs and other school-based programs were social support systems that were constructed based on the robust data related to how GSAs can provide space for empowerment and change, creating a safe space and climate for LGBTQ youth. This, in turn, can promote many positive outcomes in schools (i.e., school engagement, safety, acceptance, wellbeing) and decrease substance use, victimization, and psychological distress among other risks. Supportive and accepting peers were a system of social support that fostered higher levels of school belongingness, school engagement, academic success, sense of safety, and minimized levels of depression and school victimization for LGBTQ youth. School administrators and teachers were another system of support for LGBTQ youth. The higher the number of safe adults that were identified at school, the greater the school engagement for LGBTQ youth. Supportive adults at school, through the knowledge, resources, and connections they have about LGBTQ issues, acted against bigotry and victimization incidents at school, and fostered a positive student–teacher relationship for LGBTQ youth. Subsequently, they perceived a safer and accepting classroom environment, increased sense of school belonging, academic success, and wellbeing. School policies were constructed as a system influential to LGBTQ youth outcomes in schools. Schools with LGBTQ-inclusive policies reported lower levels of victimization, and increased sense of safety and opportunities for LGBTQ youth to act towards an empowering climate. School climate arose as an overarching system where the other systems (i.e., GSAs, school policies, curriculum, school administrators and teachers, peers) interacted to foster a safer and accepting climate for LGBTQ youth, promoting tolerance, respect, academic success, wellbeing, and school connectedness.

Based on the seven systems of social support for LGBTQ youth in schools, social support in schools can be defined as an understanding of systemic interactions amongst the seven systems (i.e., family, peers, curriculum, GSAs, school administrators and teachers, school policies, and school climate) and how each system, uniquely and in overlap, can both positively promote academic, socioemotional, and behavioural outcomes, and moderate the health and psychological risks typically associated with LGBTQ youth in schools. Studies that did not align through the lens of ecological systems focused on specific definitions of social support. For example, Day and others [27] conceptualized social support as LGBTQ youths’ perception of teachers as caring, fostering supportive classroom environments, and friendly and attentive classmates encouraging inclusivity in activities. McDonald [76] mentioned the difficulty in defining social support due to the multiple interpretations present in the literature. In their review, social support was defined as social, school, and family connectedness, support from peers, adults, advisors, and support groups [76]. Therefore, grounded in the ecological systems approach, social support cannot simply be understood in a single dimension but across multiple dimensions. This study further enhances the importance of defining, evaluating, and measuring social support for LGBTQ youth through multiple dimensions.

### Changing the narrative of social support: from passive recipients of support to opportunities and spaces for activism, skill learning, and engagement

The second objective of this review was to identify current research on outcomes for LGBTQ youth given the implementation of the social support systems. Identifying current research shed light to understand how social support provided across the social support systems are associated with LGBTQ youth outcomes. The current research on social support outcomes for LGBTQ youth sheds light on the multifaceted nature of social support systems shown to influence LGBTQ youth outcomes in schools.

The current research on family systems focuses on fostering positive connections between parents and LGBTQ youth. More specifically, current research expands beyond family acceptance and closeness as family support. Family support also entails the active support through advocacy, allyship, and communication. This finding was replicated in other social support systems where providing social support for LGBTQ youth entails the act of standing up, advocating, and challenging the LGBTQ-related issues present in schools and community.

Current research on curriculum support highlights variance in the implementation of LGBTQ-inclusive curriculum. LGBTQ-inclusive curriculum was most often incorporated in social sciences, humanities, and health classes where students were able to make authentic connections between their lives and LGBTQ-relevant social events (i.e., Stonewall, DADT legislation). Moving towards a systematic implementation of LGBTQ-inclusive curriculum that expands beyond social sciences, humanities, and health classes is an important step to provide safety for LGBTQ youth in schools. A heteronormative curriculum excludes LGBTQ youth from making authentic connections with their own lives, subsequently influencing their interest and engagement in classrooms. Increasingly incorporating LGBTQ-inclusive curriculum in education can move towards the vision for LGBTQ youth to foster authentic connections between their identity and their curriculum. This can result in improvements in their learning, wellbeing, identity exploration, and foster a supportive school and classroom climate. Like the family system, pushing for a LGBTQ-inclusive curriculum moves the system to actively challenge and disrupt the homophobia and injustice that is present in schools. Based on the findings from both systems, it appears that activism, advocacy, and this shift towards criticality against an injustice educational system is common in the literature reviewed from 2017 through 2021.

Current research on GSAs and other school programs were effective in creating a safe space for empowerment and change for LGBTQ youth. Though GSAs had different functions based on the schools’ needs and context, two of GSAs’ functions were to act as a space for advocacy and education, and acquire coping skills and resources to support their mental health. Similar to the previous systems, GSAs are moving towards providing LGBTQ youth the skills and opportunities necessary to be active participants in fostering a LGBTQ-inclusive school environment and make connections to the wider community for support.

Current research on peer support similarly highlights the importance of peers as active participants in schools to foster a sense of safety and positive classroom environment for LGBTQ youth. Beyond peers as allies, the act of peer education and intervention where peers take an active role to support their LGBTQ peers in schools led to increased sense of safety and positive classroom experiences for LGBTQ youth. Positive friendships, also known as ‘thick’ friendships, pushed LGBTQ youth to question their sexualities, reflect, and consider how their LGBTQ identity emerges in their lives. This form of close relationship with friends helped LGBTQ youth take an active role in self-reflection of their LGBTQ identity and disruption against existing oppression in schools.

Current research on school administrators and teachers focused on school staffs’ LGBTQ-inclusive knowledge, relationships with students, and opportunities for students to open the space for discussion on inclusion and diversity. School administrators and teachers have the power to create opportunities for students to foster awareness of LGBTQ issues in their schools through community events (i.e., community art gallery). This shows the importance for school administrators and teachers to have the knowledge and skills to create opportunities for students to be active participants in critical dialogue and reflection, subsequently promoting safety and acceptance in the classroom.

Like the interaction between GSAs and the community system surrounding the school, school policies were also impacted by the socio-political values of the wider community. Inclusive school policies allowed students to have opportunities to create change in schools, such as the creation of LGBTQ-inclusive events like Pride Prom and the Day of Silence to acknowledge and promote awareness of LGBTQ issues and inclusivity. Recent research further emphasized the importance of a school-wide approach to effect change in schools and incorporate inclusive policies. The research on school policies as a social support system emphasizes social support as an interaction of systems where the larger context and values can impact both the inclusivity of school policies for LGBTQ youth and the level of supportiveness from school administrators and teachers, family, and peers.

School climate, the last social support system, highlights the interrelatedness between all systems. Many articles indicated the robustness of a positive school climate and the academic, socio-emotional, and behavioural benefits for LGBTQ youth. To achieve a positive school climate, each social support system is relevant to provide social support for LGBTQ youth. Each social support system can influence each other in their effectiveness to provide the necessary space and opportunity for LGBTQ youth to act and challenge their school environment.

In sum, the current research on social support for LGBTQ youth has moved beyond understanding LGBTQ youth as passive recipients of education to recognizing LGBTQ youth as active co-creators of supportive spaces and opportunities that promote inclusive school climates that foster a sense of belongingness and safety. Other reviews exploring the impact of positive school climate on LGBTQ youth similarly emphasized the importance of a positive school climate for LGBTQ youth. Ancheta and others [2] found that a positive LGBTQ-specific school climate, as defined by supportive staff, teachers, and nurses, decreased suicidality among LGBTQ youth, promoting student empowerment and visibility through GSAs, inclusive curricula, and inclusive policies. Like previous reviews, this review highlights the importance of social support for LGBTQ youths’ experiences in schools. However, this research expands on the change in narrative that may be an indication that social support is more than providing support to LGBTQ youth. Rather, social support is changing the narrative from passive LGBTQ youth towards active LGBTQ youth, taking initiative to create change and develop skillsets to be successful in their school (i.e., both academic and social outcomes), aligned with more self-determined behaviours.

### School administration and larger community environment as barriers to supporting LGBTQ youth in educational settings

The third objective of this review was to identify barriers and inconsistencies to support LGBTQ youth in schools. Though seven social support systems were identified to foster positive socioemotional, behavioural, and educational outcomes, barriers, and inconsistencies to support LGBTQ youth were identified in each system.

Family support did not consistently buffer negative emotional, behavioural, and educational outcomes. Rather, general parental support was associated with peer victimization, self-harm, and poorer academic success [17, 90, 96]. Button [17] found that victimized LGBQ youth performed worse academically when they reported general parental support, indicating nuances between the buffer from parental support on LGBTQ youth outcomes. Inconsistencies may be explained by the functionality of family support as LGBTQ youth may perceive that their family support is ineffective to resolve harassment experienced at school.

Several studies indicated the barriers of incorporating LGBTQ-inclusive curriculum in an effective manner in schools. One aspect includes the need to understand the hidden curriculum that exists beyond the formal, academic curriculum. The social relationships and school spaces can convey heteronormativity, adversely affecting LGBTQ youth and their sense of safety and engagement in schools. Additionally, there is a need for administration to provide support for teachers to effectively incorporate LGBTQ-inclusive curriculum. Oftentimes, teachers miss teachable moments that is conducive to inclusive curriculum due to the rigid curriculum of high stakes testing and fear of parental backlash. There is indication where GSAs can be spaces used to insert LGBTQ-inclusive curriculum in an informal space to compensate the barriers that exist in classrooms (e.g., rigid curriculum). Therefore, school administrators act as key members to ensure a LGBTQ-inclusive curriculum can be incorporated effectively in classrooms.

GSAs also demonstrated barriers and difficulties in delivering positive outcomes for LGBTQ youth. Schools restricted GSA activities and presence because of sociopolitical reasons (i.e., parental and community backlash, surrounding political environment), limiting activism for LGBTQ youth. There is a need to negotiate between GSA advisors and administration for LGBTQ spaces in schools. Beyond macro-level barriers, GSAs also encountered difficulties in program implementation because of a lack of staff training to discuss sensitive topics (e.g., sexuality) in schools. There were inconsistent results in the benefits of having a GSA in schools. Possible barriers may be due to the larger school and geographic context as being involved in GSAs in more hostile or unsafe environments that place LGBTQ youth at risk. In schools and geographical areas that is more hostile, the presence and involvement of GSA-related activities is associated with increased risks of safety and decreased positive outcomes.

Peers were inconsistent in their support for LGBTQ youth. Victimized LGBTQ youth who had peers to confide to and discuss problems performed worse academically, had lower self-esteem, and had increased suicidal ideation. For racialized LGBTQ youth, peer support did not moderate perceived discrimination in schools and had no association with school performance. In addition, the lack of association for racialized LGBTQ youth suggests how peer support may be overshadowed by other (non)-LGBTQ concerns (e.g., victimization based on ethnicity, lack of family acceptance due to cultural norms and stigma towards LGBTQ identities).

School administrators and teachers were met with barriers to effectively support their LGBTQ youth in schools. Many LGBTQ youth reported not reaching out to school adults for support. The barrier appears to lie on the onus of LGBTQ youth to reach out to school staff for support. This can be due to a lack of trust or belief that teachers or administrators can effectively help them. LGBTQ youth reported teachers not knowing how to intervene in situations of harassment or teachers not feeling prepared to teach inclusive content and answer LGBTQ-related questions. LGBTQ students reported the need for teachers to increase their responsibility in teaching and conveying LGBTQ-inclusive material as the burden lies on students to provide education to their peers. From the perspective of school staff, they perceived the need for external support to coordinate support for teachers for curricular efforts, activities, and actions to reduce harassment towards LGBTQ youth and foster a greater sense of safety. Having an external coordinator as the point person to organize efforts to push the school for LGBTQ inclusion can increase LGBTQ acceptance in schools.

The wider sociopolitical context that surrounds the school has an influence on the availability of LGBTQ-inclusive school policies. The sociopolitical context act as barriers limiting the schools’ abilities to have GSAs and school staff to show support for their LGBTQ youth. However, there has been mixed evidence showing an inverse relationship between an increase of inclusive school policies and a decrease in perceptions of safety. Though there may be inclusive policies put in place in schools, such policies may not be consistently enforced by school staff, lending to the ineffectiveness of inclusive policies. Consistent implementation of inclusive school policies will require dedicated school staff to monitor the progress of policy implementation. In this review, school administrators and teachers have mentioned the need to have an external staff coordinator to monitor consistent implementation of inclusive school policies due to the lack of time and energy. School principals also played a major role in the implementation of inclusive school policies as top-down administrative support is needed to send a message to school staff that they are supported by administration should they receive family or community backlash. Compared to the findings in this review, Russell and others [97] similarly found that inclusive policies have been associated with improved school climates for LGBTQ and all youth, with LGBT students reporting feeling safer at school, hearing less verbal harassment, and experiencing less gender and sexual-specific victimization. The review emphasized that mixed results were rather due to the lack of appropriate communication of policy implementation. The findings in this review provided added evidence that it is insufficient just to simply have inclusive school policies. Rather, the implementation of inclusive school policies throughout the environment and ensuring that the policy is communicated across all relevant stakeholders is key to providing effective support for LGBTQ youth.

### A whole school approach to support LGBTQ youth with particular focus to subpopulations within the LGBTQ acronym

The fourth objective of this review was to identify areas for future research for the seven social support systems and their associations with LGBTQ youth outcomes in schools. The barriers and inconsistencies found to support LGBTQ youth across each system merits further research to explore the nuances in each system and their relationships to LGBTQ youth outcomes in school.

Within family support, there is a nuance that lie between fathers and mothers, and LGBTQ boys and girls, indicating a need to understand the complex nature of family relationships and reasons why certain family members may provide more effective social support towards LGBTQ boys or girls. For GSAs, there needs to be further exploration to understand effective methods to overcome problems in discussing gender and sexuality in school settings due in part to the lack of school staff training and student sensitivity towards LGBTQ issues. Different aspects of a GSA (i.e., presence, membership, engagement) have shown different social support outcomes for LGBTQ youth. In schools that are hostile and unsafe, positive outcomes from GSA presence and engagement dissipate, highlighting the interaction between the school climate and GSAs’ ability to be considered as an effective social support system. Mixed findings demonstrated an inverse relationship between GSA presence and lower sense of safety by LGBTQ youth. Lastly, though GSAs were primarily conceptualized as gay-straight alliances, an exploration of gender-sexuality alliances can provide insight in the nuances between experiences of students from diverse sexualities and diverse genders.

For peer systems, it is necessary to further explore reasons why peer support is either positively associated with more behavioural and emotional risks or have a lack of association. This can be due to the nature of peer support. As peer rumination can lead to further issues in schools rather than problem-solving discussions, the nature in how peers support LGBTQ peers in schools can shed light why there may be such an association. Another avenue of future research is the intersection of ethnicity among LGBTQ youth. The lack of association between peer support and positive social outcomes for racialized LGBTQ youth may be a result of the interplay of other identities that require other forms of support. For example, LGBTQ Asian youth may have an increased emphasis on the importance of family and familial piety, the need for racialized youth to bring pride to their family and minimize shame [68]. Perhaps for racialized LGBTQ youth, the lack of association between peer support and positive outcomes may be a result that lean towards family systems as increasingly important for such racialized youth.

An area of future research involves an exploration of methods to circumvent the larger sociopolitical context that limits the provision of LGBTQ support via inclusive policies. One possible avenue to provide LGBTQ support can be under the guise of Universal Design for Learning (UDL). This framework suggests the need to support all students, focusing LGBTQ support under the need to support diverse students. Another avenue of research involves exploring an explanation for inclusive policies predicting lower perceptions of safety. This may be due to the inclusive policies setting up motion to create change towards an inclusive school environment. Creating change, however, can still lead to decreased sense of safety and increased harassment issues for LGBTQ youth.

One of the specific populations highlighted to be a key support system for LGBTQ youths were educators. One constructed theme involved the inconsistency in showing support through their actions. Several of the themes highlighted how students perceived their school staff members (teachers, counselors, school psychologists, administration, principals) as being hesitant to discuss LGBTQ issues. By being hesitant and uncomfortable to teach LGBTQ issues, a norm of LGBTQ silence exists in the school environment. Attitudes and beliefs where educators believed that homosexuality and other LGBTQ topics should not be discussed in school can lead to students perceiving their school staff as uncaring and exclusive towards LGBTQ youth. Therefore, educators and other school staff members need to be comfortable and foster an inclusive attitude and belief that they are supportive of all students, as shown through their actions. Effective actions students have mentioned include consistent intervention against LGBTQ-specific harassment, and opening dialogue on the importance of inclusion and acceptance (i.e., through a LGBTQ-inclusive curriculum). When students heard LGBTQ-inclusive topics in their classes, they felt an increased sense of safety. It is therefore important to have teachers be comfortable and open to teach LGBTQ-inclusive curriculum to increase LGBTQ youths’ sense of safety.

The findings of this scoping review indicate three primary implications for future research and application. First, a whole school approach was emphasized by the themes as one of the most effective ways to provide social support for LGBTQ youths. Studies that focused on specific domains of support such as peer support or family support have similarly shown their effectiveness in supporting LGBTQ youth. However, having all relevant stakeholders involved in the process of supporting LGBTQ youth, such as a whole school approach, was evidently the most effective. Notwithstanding, a collaborative, whole school approach may be overly idealistic and an unrealistic approach for schools embedded in a larger, more conservative environment. A middle ground between realism and idealism could be attained by auctioning the GSA as a physical, supportive space where students can feel safe (within the club). This would be contrasted to having GSAs be a space for education and activism towards an increasingly LGBTQ-inclusive environment. In some cases, the inclusion of GSA spaces within schools may mean that, generally, the larger school spaces are unsafe for LGBTQ youth, influencing the concrete actions that educational stakeholders can take to provide support and opportunities for their students.

Second, there were differences in perceived support and outcomes depending on the subpopulation of LGBTQ youth, highlighting the issues of generalizing the LGBTQ youth population as a homogenous population. For example, there were different perceptions of safety and struggles between sexual orientation minority youth and gender identity minority youth. For example, there were unique issues of gender nonconformity for youth who did not conform to their assigned gender at birth, whereas LGBQ youth were faced with victimization due to their sexual orientation. Of note, there have been a recent trend for studies reviewed between 2017 through 2021 to include trans and gender non-conforming (TGNC) youth as the focus, beyond the LGBTQ general identities. As several key findings foregrounded sexual identities over gender identities, future inquiries of LGBTQ youth should take into consideration the specific LGBTQ subgroups to be studied by researchers. Particularly, intersectionality should be taken into consideration as issues of gender, class, and ability may influence how specific LGBTQ students experience school supports. By doing so, researchers can be aware of the various intersecting sexual orientation and gender identities that LGBTQ youth manage and to be inclusive of programs, interventions, and strategies that are intended to support LGBTQ youth as a whole. Though the seven social support systems have been shown to effectively support LGBTQ youth, the inconsistencies that some articles brought up shed light with the interaction of these social support systems and their intersectional identities.

Third, there were differences in perceived support and outcomes depending on the ethnicity and race of the youths. For example, LGBTQ Eurocentric youths experienced increasing emotional and behavioral distress due to LGBTQ-specific victimization, whereas LGBTQ racial and ethnic minority youths experienced less distress. A hypothesis explaining the difference may be linked to the coping skills and resilience that the racial and ethnic minority LGBTQ youths have already learned to cope in the face of racial and ethnic-specific victimization. This results in more frequent use of their coping skills and a higher resilience and grit in the face of LGBTQ-specific victimization and being in a school environment that is perceived to be less safe. Therefore, future inquiry should consider the multiple, intersecting minority identities LGBTQ youths may have had to juggle and its effect on their perceived safety and support in their school environment. Particularly, critically thinking through race and its impacts on the experience of school supports for LGBTQ youth should be a priority for future research. Based on their intersectional identities and experiences in schools, the seven social support systems found in this review may vary in effectiveness based on their other identities.

## Limitations

This scoping review attempts to consolidate material from 2007 through early 2021, organize, and respond to the four research questions of defining social support, identifying the current social support outcomes for LGBTQ youth, the barriers and inconsistencies encountered by the social support systems, and the areas for further research because of the barriers and inconsistencies found in the literature. Due to the scope of the review, the literature search strategy was broad and resulted in a larger volume of articles. Though the search strategy was comprehensive, consulting various experts to ensure rigidity and confidence of the search strategy, a scoping review search strategy utilizes a less defined search compared to a full systematic review. Additionally, difficulty in consolidating a comprehensive search term strategy can lead to an increasingly narrow understanding of LGBTQ individuals. For example, no articles explored nonbinary parents or children. The search strategy did not account for an intersectional understanding of LGBTQ+ identities, particularly Two-spirit (2S) identities. As the initial search strategy was executed in 2017, Two-spirit identities were uncommon and, subsequently, not taken into consideration. Future reviews should include the search strategy to account for an intersectional approach to the 2SLGBTQ+ community. Further, search strategies for LGBTQ+ terms are difficult to standardize and capture comprehensively. Even though various experts were consulted to ensure confidence in the search strategy, the rigidity of keywords and subheadings in article search engines can be barriers in capturing the nuances inherent to the 2SLGBTQ+ community. Particularly, transgender and non-binary (TGNC) terms were limited. Moving forward, it would be important to implement the University of Minnesota’s search hedges (tested and standardized search strategies to retrieve articles on specific concepts) for TGNC terms [115]. As well, this scoping review did not follow the required critical appraisals and risk of bias assessment found in systematic literature reviews. However, based on the purpose of this study, a scoping review methodology was the best approach due to the wide body of literature that has not been comprehensively reviewed. Additionally, scoping reviews are best used when the purpose is to clarify working definitions (i.e., social support) and conceptual boundaries of this topic (i.e., social support systems for LGBTQ youth in schools), and identify gaps in existing literature. Therefore, though a scoping review utilizes a less defined, broader search strategy, resulting in a broader literature less systematic and confident compared to a full systematic review, the purposes of this study and research questions align with the scoping review design.

## Conclusion

There have been many studies replicating the risks that LGBTQ youth experience in schools: socioemotional (e.g., depression), behavioural (e.g., substance use), and educational (e.g., truancy, decreased school engagement). However, literature exploring the positive or protective factors for LGBTQ youth has been steadily increasing in the field of youth and LGBTQ studies. As it is still an emerging perspective to explore the protective factors for LGBTQ youth through positive youth development lens, this review consolidated literature and gave rise to an organizational framework to consolidate the various systems of social support for LGBTQ youth in schools. From the review, social support consists of seven social support systems (family, curriculum, school professionals and teachers, peers, school policies, GSAs and programs, and school climate) that, both uniquely and in overlap, are positively associated with the promotion of positive socioemotional, behavioural, and educational outcomes, as well as the moderation of the risks typically associated with LGBTQ youth in schools. Though the literature consistently revealed the benefits of ensuring that these seven systems are present to positively support LGBTQ youth’s development, inconsistencies and barriers in providing positive outcomes for LGBTQ youth was a result of (1) a lack of training and support for school administrators and teachers to enforce LGBTQ-inclusive policies and curriculum, (2) a larger sociopolitical context impeding or preventing LGBTQ activism and support in schools, and (3) unique differences within the subgroups of LGBTQ youth including ethnicity, sex, and gender identity and expression. Future research should explore the gaps present in this review to address the barriers and inconsistencies found to effectively provide social support for LGBTQ youth across these seven systems. This review highlights a positive outlook towards the available systems of social support to promote positive development for LGBTQ youth. Though the literature has been clear surrounding the risks associated with LGBTQ youth, this scoping review endeavored to provide a positive outlook on LGBTQ youth’s school experiences by highlighting how these systems of social support allow LGBTQ youth to act as active participants in the promotion of a positive and safe school climate.

## Supplementary Information

Below is the link to the electronic supplementary material.

Supplementary file1 (DOCX 65 KB)
