# Supplementary material for: Social support in schools and related outcomes for LGBTQ youth: a scoping review
Source: Discov Educ. 2022 Nov 14;1(1):18. doi: 10.1007/s44217-022-00016-9 (PMC9662773; doi:10.1007/s44217-022-00016-9)
Supplement: Supplementary file 1 — Supplementary file1 (DOCX 65 KB) [file 44217_2022_16_MOESM1_ESM.docx]

## **Appendix**

### Table 1

### *Keyword Search across Five Databases*

| **First key concept**  **(LGBTQ+)** | **Second key concept**  **(School)** | **Third key concept**  **(Social Support)** |
| --- | --- | --- |
| **PsycINFO Subheadings**  Homosexuality or (attitudes towards) or Male Homosexuality  Bisexuality  Transgender or (attitudes towards)  Gender Identity  Lesbianism  Hermaphroditism  Sexual Orientation  Transsexualism  Gender Identity Disorder  Sexuality | **PsycINFO Subheadings**  High School  Middle Schools  Elementary Schools  Junior High Schools  Secondary Education  Religious Education  Public school education  Private school education  Student attitudes  Teacher attitudes  Boarding schools  Charter schools  Military schools  Technical schools  High school students  Elementary school students  Junior high school students  Elementary education  High school education  Middle school education  Nontraditional education  Vocational education/vocational | **PsycINFO Subheadings**  Peer relations  Social groups  Clubs (Social Organization)  Social support  Social programs  After School Programs  Extracurricular activities  Group Intervention  Online social networks  Social networks  Community involvement  Therapeutic social clubs  Peers  Support groups  Activism  Student Activism |
| **ERIC Subheadings**  “Homosexuality”  “Lesbianism”  “Bisexuality”  "Sexual orientation"  "Gender issues"  "Sexual identity"  "Sexuality" | **ERIC Subheadings**  "Ancillary school services"  "Bilingual schools"  "Boarding schools"  "Catholic schools  "Charter schools"  "Community schools"  "Comprehensive school health education”  "Consolidated schools"  "County school districts"  "Day schools"  "Disadvantaged schools"  “Elementary Schools”  “Elementary school students”  "School districts"  “High School students”  “High school”  “Middle School Students”  “Middle Schools” | **ERIC Subheadings**  "Peer acceptance"  "Peer coaching"  "Peer counseling"  "Peer mediation"  "Peer relationship"  "Social justice"  "Social promotion"  "Social services"  "Social support groups"  "Clubs"  "Group activities"  "Group counseling"  "Group therapy"  "Groups"  "Community support"  "Activism"  "School activities"  "School community programs"  "School guidance"  "School organization"  "School safety"  "School space"  "School support" |
| **GenderWatch Keywords**  “Gays & lesbians”  GLBT Studies  “Gender identity”  “Sexual orientation”  “Transgender persons”  Gender nonconforming children and adolescents  Lesbian, gay, bisexual, and transgender youth  “LGBTQ studies”  Sexuality  “Social identity”  Homosexuality  “Gender differences”  Bisexuality  Gender equity  Transgender  Lesbianism  “Gender identity disorder”  Hermaphroditism  Transsexualism | **GenderWatch Keywords**  “Elementary education”  “Rural school”  Rural education  “Secondary school”  “High school”  “Middle school”  “Elementary school”  Charter schools  School district  “Public school”  “Primary school”  “Catholic school”  “Junior high school”  “Secondary education”  “Boarding school”  “High school education”  “Vocational education”  “Vocational school students” | **GenderWatch Keywords**  “Education policy”  “Social structure”  Activism  “Social justice”  “Sex education”  “Health education”  “Educational partnership”  “Gay-straight alliance”  Alliances  Clubs  “Peer relations”  “Political activism”  “Education reform”  “Equal rights”  “Social support”  “Inclusive education policy”  “Student voice”  “Social connectedness”  “Sexual health education”  “Straight allies”  “Youth program”  “School-based prevention”  “After school program”  “Extracurricular activity”  “Social network”  “Community involvement”  “Support group”  “Policy making”  “Safe school”  “School safety” |
| **ProQuest Dissertations and Theses Subheadings**  “Homosexuality”  “Lesbianism”  “Bisexuality”  "Sexual orientation"  "Gender issues"  "Sexual identity"  "Sexuality" | **ProQuest Dissertations and Theses Subheadings**  "Ancillary school services"  "Bilingual schools"  "Boarding schools"  "Catholic schools  "Charter schools"  "Community schools"  "Comprehensive school health education”  "Consolidated schools"  "County school districts"  "Day schools"  "Disadvantaged schools"  “Elementary Schools”  “Elementary school students”  "School districts"  “High School students”  “High school”  “Middle School Students” “Middle Schools” | **ProQuest Dissertations and Theses Subheadings**  "Peer acceptance"  "Peer coaching"  "Peer counseling"  "Peer mediation"  "Peer relationship"  "Social justice"  "Social promotion"  "Social services"  "Social support groups"  "Clubs"  "Group activities"  "Group counseling"  "Group therapy"  "Groups"  "Community support"  "Activism"  "School activities"  "School community programs"  "School guidance"  "School organization"  "School safety"  "School space"  "School support" |
| **Web of Science Keywords**  Same-sex  Homosexual*  Bisexual*  Gay  Lesbian*  Transgender  LGBT*  "Sexual Orientation"  Gender-role  "Gender expression”  "Sexual minority"  Nonheterosexual  "Diverse sexualities"  Heteronormativity  Transsexual  “Gender identity” | **Web of Science Keywords**  "High school"  "Secondary school"  "Primary school"  "Catholic school"  "Middle school student"  "High school student"  "Elementary school student”  Primary/elementary  "Middle school"  "Elementary school"  "Junior high school"  "Secondary education"  "Boarding school"  "Charter school"  "Institutional school"  "Elementary education"  "High school education"  "Vocational education"  "Vocational school student" | **Web of Science Keywords**  "Peer relations"  "Social support"  "Sex education"  "Inclusive education policy"  "Student voice"  "Social connectedness"  "Gay-straight alliance"  "Sexual health education"  "Straight allies"  "Youth program"  "Psychological empowerment”  "School club"  "School-based prevention"  Activism  "Multicultural education"  "Social group"  "After school program"  "Extracurricular activity"  "Group intervention"  "Online social network"  "Social network"  "Community involvement"  "Support group"  "School safety"  "Educational Program"  "Safe school" |

*Note subheadings were used when possible pending search options in each database. Otherwise, keywords in quotation marks were used to show specific keywords used for the search. Each column in each database was then combined with the Boolean term ‘AND’, with each keyword under each key concept combined with the Boolean term ‘OR’.*

*Phase Two:* Full text screening

(*n* =94)

Excluded articles for general findings tangentially related to social support for LGBTQ students

(*n* = 90)

*Phase One:* Titles and abstracts screening

(*n* = 184)

Excluded articles on non-LGBTQ specific population, unrelated to school environment, social support outcomes not for students, were not in English

(*n* = 349)

Duplicates removed

(*n* = 533)

*Search Strategy:* Literature search databases (PsycINFO, ERIC, GenderWatch, ProQuest Dissertation, Web of Science)

(*n* = 565)

Final sample

**94 articles**

### *Figure 1. Visual flowchart of search strategy*

### Table 2

### *Study Characteristics of 94 Articles (Study Type, LGBTQ Acronym, Participant Sample Range, School Setting Type[Educational Level], Types of Social Support)*

| **Study Type (*n* = 94)** | ***n*** | **%** |
| --- | --- | --- |
| Quantitative | 48 | 51.06% |
| Qualitative | 43 | 45.74% |
| Mixed Methods | 3 | 3.19% |
| **LGBTQ Acronym* (*n* = 102)** |  |  |
| LGBTQ/GLBTQ | 40 | 39.22% |
| LGBT/GLBT | 15 | 14.71% |
| Sexual minority/SMY | 10 | 9.80% |
| LGBQ | 7 | 6.86% |
| LGB/GLB | 6 | 5.88% |
| Transgender/Trans*/TGD | 5 | 4.90% |
| GM/Gender-variant | 4 | 3.92% |
| GSM/GSD | 3 | 2.94% |
| SSA | 3 | 2.94% |
| LGBTQQ | 2 | 1.96% |
| LGBTQ2S | 2 | 1.96% |
| LGBTQ+ | 2 | 1.96% |
| Bisexual/Pansexual | 1 | 0.98% |
| MSMY (multi-ethnic) | 1 | 0.98% |
| Queer | 1 | 0.98% |
| **Participant Sample Range (*n* = 94)** |  |  |
| Grades 6 – 12 | 2 | 2.13% |
| Grades 7 – 12 | 10 | 10.64% |
| Grades 8 – 12 | 6 | 6.38% |
| Grades 9 – 13 | 20 | 21.28% |
| Grades 10 – 12 | 4 | 4.26% |
| College | 3 | 3.19% |
| High school/College | 5 | 5.32% |
| Elementary School | 4 | 4.26% |
| Elementary/High School | 1 | 1.06% |
| High school | 16 | 17.02% |
| Middle school | 4 | 4.26% |
| Middle school/high school | 7 | 7.45% |
| Parents/advisors/adults | 5 | 5.32% |
| Not specified | 7 | 7.45% |
| **School Setting Type – Educational Level (*n* = 94)** |  |  |
| High school | 46 | 48.94% |
| High school/Middle school | 28 | 29.79% |
| Elementary/Middle/High school | 4 | 4.26% |
| Middle school | 3 | 3.19% |
| Elementary school | 4 | 4.26% |
| Community center | 1 | 1.06% |
| College/University | 3 | 3.19% |
| Not specified | 5 | 5.32% |
| **School Setting Type – School Type (*n* = 94)** |  |  |
| Private school | 5 | 5.32% |
| Catholic school | 4 | 4.26% |
| Alternative school | 2 | 2.13% |
| Democratic school | 1 | 1.06% |
| Independent school | 1 | 1.06% |
| Not specified | 81 | 86.17% |
| **Types of Social Support* (*n* = 188)** |  |  |
| Peer Support | 24 | 12.77% |
| Parental Support | 16 | 8.51% |
| Community Support | 9 | 4.79% |
| School Support (*n* = 139) |  |  |
| Gay-Straight Alliance | 42 | 22.34% |
| Supportive Staff | 34 | 18.09% |
| Supportive Teachers | 24 | 12.77% |
| Positive School Climate | 12 | 6.38% |
| Programs and Policies | 11 | 5.85% |
| School-Wide Approach | 9 | 4.79% |
| Curriculum | 7 | 3.72% |

**Each study mentioned multiple types of social support within their studies, therefore not totaling to 54 types of social support.*

### Table 3

### Thematic Table of 94 Articles

| **Constructed Themes** | **Unique Theme Source** | **Thematic Elements** |
| --- | --- | --- |
| **Family** | | |
| High actual or perceived family/caregiver support buffered many negative social-emotional or educational outcomes | 12 | 1. When actual or perceived family/caregiver support was low, levels of emotional and behavioral distress (suicidal ideation and school difficulties) were high **(Antonio and Moleiro, 2015; Pearson and Wilkinson, 2013)** 2. Significantly higher levels of psychological distress were found among high school graduates who reported LGBT harassment at home **(Bidell, 2014)**. 3. Particularly for LGBT homeless youth, family-based harassment is more psychologically distressful compared to school-based harassment **(Bidell, 2014)** 4. Low caregiver support, uniquely and in combination with peer victimization and a sexual minority identity were associated with higher levels of depressive symptomatology for SM youth **(Johnson et al., 2011)** 5. Family acceptance enabled LGBT students to evolve socially and engage in cultural action to help them be strong and advocate, which promoted positive outcomes, including a safe space for students to share experiences, reflect, raise consciousness, and enable praxis to foster a positive school environment for LGBT students **(Grace and Wells, 2009; Wright et al., 2012)** 6. Particularly for MSMY, increased family support was associated with better school performance **(Craig and Smith, 2014)** 7. Family support is significantly associated with reduced risk of victimization, substance use, and suicidality for students, regardless of sexual orientation **(Button et al., 2012)** 8. Experiencing sexual stigma makes SM/SSA youth perceive less parental closeness and support, therefore being at an increased risk of experiencing depressive symptoms, substance use, running away from home **(Johnson et al., 2011; Pearson and Wilkinson, 2013)** 9. Adolescents' perceptions of closeness with their parents, their involvement in shared activities and their overall sense of support from their families were associated significantly with their well-being and risk behaviors **(Pearson and Wilkinson, 2013)** 10. For LGBTQ youth, working with parents to foster youth resilience moderated the effects of general victimization on suicidality and greater school belonging and indirectly predicted lower educational concerns **(Poteat et al., 2011)** 11. LGBT-affirming resources aimed at developing family support fostered student academic well-being and physical and emotional safety **(Craig et al., 2018)**. 12. Parent advocacy and allyship was important for transgender children to feel safe and be their authentic selves in their classroom **(Goldstein et al., 2018)**. 13. Parental communication and parental trust was shown to minimize homophobic victimization **(Pace et al., 2020)**. |
| Family/caregiver support is not consistently adequate to buffer the negative emotional, behavioral, and educational outcomes | 3 | 1. Victimized LGBQ youth who feel supported by their parents are more likely to perform poorly in school **(Button, 2016)** 2. When LGBTQ youth were victimized, general parent support did not consistently attenuate the negative outcomes **(Button, 2016; Poteat et al., 2011)** 3. Family support may be protective against violence victimization and self-harm among high school students, however, effects were less robust for GM youth **(Ross-Reed et al., 2019)** |
| Sex differences within family experiences arose highlighting complexities of family/caregiver support | 3 | 1. Overall sense of support from family is more important than parental closeness or involvement to explain the decreased well-being among SM girls. For SM boys, involvement in shared activities with parents is associated with an increased likelihood of risk behaviors, including substance use and running away from home **(Pearson and Wilkinson, 2013)** 2. (SSA) Adolescents who reported less disclosure to their fathers (only fathers not mothers) experienced less acceptance from peers, felt more peer role strain, and reported lower levels of self-esteem, higher levels of depression, and lower levels of school identification/belonging **(Bos, et al., 2008)** 3. For SM girls, no association was found between caregiver support and peer victimization **(Johnson et al., 2011)** |
| **Curriculum** | | |
| LGBTQ-inclusive curriculum was most often taught in social sciences, humanities, and health classes and fostered authenticity with students, creating an inclusive classroom | 6 | 1. By exploring conceptual connections across sociology classes, inquiring key historical events such as Stonewall and the rise of Harvey Milk or DADT legislation allowed students to make more authentic connections between their lives and the "official" social studies topics being taught **(Mayo, 2013b)** 2. They were most often taught in social sciences, humanities, and health classes and brought opportunities for critical conversations about systematic oppression and were able to learn how such curriculum reflected their identities and created a supportive school climate, improving their learning and well-being, and fostered an authentic connection with the teacher in such classes **(Blackburn, 2007; Snapp et al., 2015a; Wright et al., 2012)** 3. A [Q]SP invites educators to work and tinker along the lives and edges of LGBTQ2 children and youth, disrupting homophobia, injustice, and other forms of oppression by centering the queer in culture **(Wargo, 2019)**. 4. Teachers agreed on the importance of weaving social justice topics in the curriculum to model critical literacy in creating an inclusive curriculum to benefit all students **(Pearce & Cumming-Potvin, 2017)**. |
| LGBTQ-inclusive curriculum led to decreased actual/perceived bullying and social-emotional outcomes (e.g., depression) and increased sense of safety for LGBTQ students. | 5 | 1. LGBTQ-inclusive curricula were associated with higher reports of safety at the individual and school levels, and lower levels of bullying at the school level. Supportive curricula were related to feeling safer and awareness of bullying at the individual and school levels and combat feelings of isolation and depression **(Luecke, 2011; Snapp et al., 2015c**) 2. School safety was higher for schools in which more students reported the presence of LGBTQ-inclusive sexuality and health education. Further, in schools where sexuality and health education was perceived as supportive of LGBTQ people and issues, there was more safety and less bullying. Schools are more personally safe and have less bullying when curricula are supportive **(Snapp et al., 2015c)** 3. When students report inclusion of LGBTQ issues in the curriculum, have accessible information related to LGBTQ issues, and when teachers intervene in sexual orientation- and gender nonconformity-based harassment, they perceive their schools as safer for gender nonconforming male students **(Snapp et al., 2015c; Toomey et al., 2012)** 4. Safety and acceptance requires the effort of the teacher to be present and update the curriculum to be inclusive to validate LGBTQ students' experiences **(Shelton & Lester, 2018)** 5. Developing a [q]ulturally sustaining stance and disposition to center SOGI issues in elementary school spaces can recognize the whole person, bringing a possible avenue to bring LGBTQ2 inclusive topics in early childhood and elementary classrooms to disrupt homophobia, injustice, and other forms of oppression benefitting LGBTQ2 children and youth **(Wargo, 2019)** |
| A hidden, heteronormative curriculum (e.g., heteronormative academic content, social spaces, relationships) exists behind the official academic curriculum that impedes LGBTQ+ youth support and engagement | 4 | 1. The 'hidden' heteronormative curriculum directly and adversely affects SM youth on their psychological and educational levels and that LGBTQ-inclusive curriculum needs to take into account not only the academic, official curriculum, but also the social curriculum (relationships) and campus curriculum (group space; **Castro and Sujak, 2014)** 2. GSAs also supplement positive educational outcomes as formal education lacks a queer perspective and content on queer history **(Lapointe, 2014; Mayo, 2013b; Woolley, 2012)** |
| There is a need for teachers to feel supported to teach LGBTQ-inclusive curriculum effectively, including the need for increased understanding and implication of LGBTQ-related content in curriculum and support from administration | 4 | 1. Teachers often missed teachable moments conducive to inclusive curriculum **(Luecke, 2011; Snapp et al., 2015a)** 2. The administration’s role was highlighted not only in a supporting capacity, but more significantly, as a catalyst for small but positive changes in the curriculum, providing the support needed for teachers **(Liboro et al., 2015)** 3. Teachers mentioned difficulty fostering an inclusive curriculum due to rigid curriculum, high stakes testing, parental resistance, and fear from parental backlash **(Pearce & Cumming-Potvin, 2017)** |
| **School programs (such as GSAs)** | | |
| Gay-Straight Alliances (GSAs) have been shown to foster a space for empowerment and change, creating a safe space and climate for LGBTQ students, subsequently leading to various positive outcomes and reduced negative outcomes | 25 | 1. GSAs help students act together to create cultural and institutional change and can be transformative for school culture and students to recuperate, engage and resist oppression, share experiences, reflect, raise consciousness, and motivate engagement in social advocacy, leadership, and create plans of actions.These spaces provide a positive physical and intellectual space where students could discuss LGBTQ issues that were silenced or omitted in the larger school community **(Elliott, 2016; Grace and Wells, 2009; Lapointe, 2014, 2015; Liboro et al., 2015; Mayberry et al., 2013; Mayo, 2013a; Russell et al., 2009; St. John et al., 2014; Woolley, 2012)** 2. The club gives students who identify a safe place to go, to have a safe place to be accepted no matter what and that it helps educate other people because people need to learn more about us **(Liboro et al., 2015).** 3. GSAs were the third space that promoted learning and social change where teachers could also participate **(Mayo, 2013a).** 4. Students who attended a high school with a GSA reported significantly more favorable outcomes related to school experiences, alcohol use, and psychological distress, and attenuating a range of health risks **(Heck, 2014; Heck et al., 2013; Poteat et al., 2013)** 5. GSA participation in activities and presence of a GSA was positively associated with school belongingness and GPA. GSA membership is also positively associated with school belongingness.1) Presence of a GSA, 2) participation in a GSA, and 3) perceived GSA effectiveness promoted school safety differently for young adult well-being and differently moderated the negative associations between LGBT-specific school victimization and well-being **(Toomey and Russell, 2013; Toomey et al., 2011)** 6. Integrating mental health promotion programs within a GSA was feasible and students found such programs enjoyable, helpful, and feasible, providing coping skills and resources **(Heck, 2015)** 7. Entering GSA classrooms offered visibility, positive symbols of acceptance, respect, and affirmation in safe spaces from homophobia/transphobia **(Bain & Podmore, 2020)** 8. Presence of GSAs was associated with lower levels of safety **(De Pedro et al., 2018)**. 9. GSAs were shown to be high functioning for LGBTQ students particularly in schools with a negative school climate, as homophobic bullying was least frequent **(Ioverno & Russell, 2021)**. 10. Schools with GSAs were shown to reduce SGM-specific and intersectional identities victimization and had higher perceptions of school safety and increased academic success **(Lessard et al., 2020)** 11. Rainbow group enabled trans students to explore, contest, and perform their gender identities in their schools, going against the binary hierarchy embodied in schools and challging the wider school culture **(McGlashan & Fitzpatrick, 2017, 2018)** 12. GSAs provided a trusting foundation to fulfill LGBTQ student needs and acts as a source and symbol that LGBTQ youth needs to have a sense of safety **(Porta et al., 2017)** 13. GSAs brought attention to exclusive policy and practices by hosting queer-friendly events, working towards a safer environment for transgender students **(Sutherland, 2019)** 14. Engaging with GSAs enabled students to form their own identities grounded in empowerment rather than as victims **(Elliott, 2016; Russell et al., 2009)** |
| GSAs can create opportunities for connection for LGBTQ students in their community | 13 | 1. GSAs can provide accountability, support, community, increased GPA, decreased feelings of isolation by connecting youth with other LGBTQ community members, events, and resources, leading to increased validation and normalization of identity, sense of hope, greater self-esteem, greater appreciation for self and other peers, adaptive social relationship negotiation skills, acceptance, and a sense of safety and empowerment to its members **(Elliott, 2016; Grace and Wells, 2009; Heck et al., 2013; Liboro et al., 2015; Mayberry et al., 2013; Mayo, 2013a, 2013b; McCormick et al., 2015; Russell et al., 2009; St. John et al., 2014; Toomey and Russell, 2013)** 2. GSAs allowed for connections to community organizations, fostering activism opportunities, connections and visibility for students **(Bain & Podmore, 2020)** 3. GSAs provides a gateway to wider LGBTQ community, supportive adults, and community resources **(Porta et al., 2017)** |
| GSAs allowed for engagement and youth involvement in schools and GSAs, leading to positive outcomes (e.g., self-efficacy). | 5 | 1. Participating in GSA was shown to be a safe space for bi/pansexual youths to 1) self-express and have pride and 2) learn and educate through advocating beyond the fe/male binary. GSA was a space for many students for self-discovery and validate their self-identity, providing the space for activities and discussion **(Lapointe, 2017)** 2. Students' involvement in transgender-related discussions in GSAs contributed to their self-efficacy to address transgender issues, particularly for those who had more GSA engagement and discussions in their GSAs **(Chong et al., 2019)**. 3. There was a positive relationship in schools with GSAs where GSM students were found to be more engagement and had better academic success **(Hazel et al., 2019)**. 4. GSA members who had higher levels of involvement and received more social-emotional support and informational resources from GSAs reported greater general civic engagement, higher levels of hope, more advocacy and awareness-raising efforts related to SOGI issues, and reduced effects of victimization **(Poteat et al., 2018, 2020)**. |
| GSAs vary in their function (e.g., advocacy, educational, socialization) | 7 | 1. GSAs were more distinct from one another on advocacy than socializing. For youths who were more actively engaged in the GSA as well as GSAs whose youth collectively perceived greater school hostility and reported greater social justice efficacy did more advocacy. The varying functions of GSAs depend on the internal provisions of support, to visibility raising, to collective social change **(Mayberry et al., 2013; Poteat et al., 2015; Woolley, 2012)** 2. Students who were more involved in accessing information/resources and in advocacy in their GSA discussed more health-related topics, prepared more awareness-raising campaigns, and had increased engagement in their school and increased knowledge gathering **(Poteat et al., 2017)** 3. GSAs where the focus was more on support/socialization less frequently integrated mental health discussions during their meeting **(Poteat et al., 2017)** 4. GSAs had distinct purposes in assisting differnet aspects of LGBTQ+ students: 1) advocacy, education, and social support; 2) litearture to reflect lives and experiences in students; 3) developing skill sets to assist students in fostering inclusion and acceptance **(Underhill, 2017)** 5. Advisors believed the primary role of the GSA was to provide an emotionally safe environment for LGBT students to bring awareness and take action, whereas students perceived fostering a sense of community and belongingness as importance, therefore GSAs serving two diverging roles **(Lapointe, 2015; Mayberry et al., 2013; Robertson, 2008)** |
| GSA's encounter challenges in their execution and in delivering positive outcomes | 15 | 1. Program implementations within GSAs to support LGBTQ+ youths had common problems which were: 1) lack of staff training and safe staff, 2) lack of student understanding of or sensitivity to LGBTQ issues, and 3) challenges of discussing sexuality in a middle school setting **(Horowitz and Itzkowitz, 2011; Liboro et al., 2015)** 2. GSAs still struggle to subvert the heteronormative school climates among other students and teachers when antigay comments occurred in the school setting or in schools where the greater community is unsafe. **(Lapointe, 2015; Mayberry et al., 2013)** 3. High schools have concerns and restrict policies on GSA student behaviors, limiting the activities that were feasible or imaginable among participants and activism was not always allowed or done **(Elliott, 2016; Fetner et al., 2012).** 4. Only 19.1% youth reported their high school having a supportive LGBT club such as a GSA **(Bidell, 2014)** 5. GSA effects were nonsignificant for general or homophobic victimizaiton, grades, and school belonging. Presence of a GSA did not predict student school engagement. However, GSA size, visibility and perceived support predicted engagement and engagement is a significant predictor of GPA. Personal involvement in a GSA did not predict student school engagement **(Poteat et al., 2013; Seelman et al.,2012, 2015)** 6. The positive benefits of GSA-related social justice involvement and the presence of a GSA disspate at high levels of school victimization **(Toomey et al., 2011; Toomey and Russell, 2013)** 7. Being in a GSA space can lead to emotional vulnerabilities to wider school community **(Bain & Podmore, 2020)** 8. GSA-admin were required to negotiate the LGBTQ2S space in school, going against indifferent culture towards supporting LGBTQ2S students **(Bain & Podmore, 2020)** 9. Presence of a GSA was not associated with mental health outcomes or predictor of help-seeking behaviors **(Colvin et al., 2019)** 10. The impact of GSA's on LGBTQ safety and school climate may vary widely across schools and geographic contexts as GSAs in rural areas may negatively related to safety **(De Pedro et al., 2018).** |
| School-based interventions (non-GSAs) were shown to be effective to support LGBTQ youth | 6 | 1. An intervention was conducted in a classroom to accept individual differences in a positive manner through active and cooperative group participation, express feelings in a positive manner shown by open discussion of emotional issues within group, and to show progress on accepting constructive feedback in a positive manner. Intervention showed an openness to address important and sensitive issues among the students. Interventions that were effective framed uniqueness as a strength and fostering a change in the classroom climate towards acceptance **(Luecke, 2011; Robertson, 2008)** 2. Youth-led theater and dialogue-based interventions was an effective strategy to address heterosexism and genderism in schools. Participation in the performance was significantly associated with increased reports of willingness to advocate for social jusitce, fairness, and equality for LGBTQQ people, increasing students' intentions to participate in macro-level change around LGBTQQ issues **(Wernick et al., 2016)** 3. There is a need to employ a pragmatic approach and focus on student safety to gain administrative support to conduct interventions **(Lassiter and Sifford, 2015; Liboro et al., 2015; Robertson, 2008)** 4. The art gallery prompted adults in the community to consider gender issues and led them to take action and behave differently, with them being more supportive or affirming of LGBTQ youth moving forward, considering the use and impact of language related to gender, and reflect on and confront their own biases related to sexuality and gender as well as confronting bias within others **(Hall et al., 2018).** |
| **Peers/Friends** | | |
| Fostering peer support and acceptance relates to lower levels of emotional and behavioral distress and fosters positive psychological, social, and educational outcomes (e.g. sense of belonging) | 13 | 1. Adolescents with higher levels of peer acceptance and lower levels of peer role strain experienced lower levels of depression and is associated significantly with their self-esteem **(Bos, et al., 2008)** 2. Higher levels of sense of belonging to GLB youth groups were associated with higher levels of school, teacher, and peer connectedness. School and teacher connectedness played an indirect role in predicting depressive symptoms, via peer connectedness. Belonging to a GLB youth group increased feelings of confidence and sense of belonging and foster expectations of being accepted by teachers and broader school community **(McLaren et al., 2015)** 3. Older adolescents were found to have less homophobic attitudes and more willing to remain friends and attend school with GL peers (**Toomey et al., 2012)**. 4. Additionally, straight allies play an important role to address anti-gay stereotypes by being an ally **(Lapointe, 2014)** 5. Being out to more friends solely or in combination with other groups of individuals (such as family) was generally associated with higher grades and less school harassment, however, youth who reported being out at home but not at school reported the worst grades and more harassment **(Watson et al., 2015)** 6. A lower quality of peer support/relationship resulted in higher levels of depression and lower levels of self-esteem and school identification **(Bos, et al., 2008)** 7. Peers were also mentioned as support systems, particularly for youth with rejecting families **(Craig et al., 2018)**. 8. Peer education and interventions have been shown to be associated with more positive school/classroom experiences and higher levels of safety for LGBTQ youth **(De Pedro et al., 2018; Fantus & Newman, 2021)** 9. Thick friendships were shown to help encourage LGBTQ youth to question their sexualities, reflect with each other surrounding questions of sexuality, and how queer positivity emerges from such rellationships, interactions, and attachments, disrupting school bullying and listening to LGBTQ youths' stories and narratives **(Gilbert et al., 2019)**. 10. For SM Chinese adolescents, having supportive peer relations mediated the effect of their suicidal behavior **(Huang et al., 2018)** 11. TGD students also mentioned the importance of having peer support in their schools, shown to have improved academic success **(Jones, 2017)**. 12. Students reported feeling encouraged seeing 'out' peers in their classrooms as a form of feeling safer in their school **(Palkki & Caldwell, 2018)** 13. Degree of safety and acceptance required peers be present to think, exist, and accept outside gender-established boxes **(Shelton & Lester, 2018)**. |
| Limited and poorly developed conclusions regarding peer support | 4 | 1. For MSMY youth, peer support was not associated with better school performance and did not moderate effects of perceived discrimination **(Craig and Smith, 2014)** 2. For LGBQ youth who have been victimized, those who had peers to discuss problems related to substance use and/or sex were more likely to perform poorly in school and report considering planning and/or attempting suicide **(Button, 2016)** 3. Positive correlation between peer support and NSSI among all students, although the relationship between peer support and NSSI was stronger among GM students **(Ross-Reed et al., 2019)** 4. Social support did not buffer the effect of victimization on self-esteem in either cisgender LGBQ students or transgender students **(Taylor et al., 2020)** |
| **School professionals, administrators, teachers** | | |
| Having a high level of within-school adult support result in positive benefits (e.g. increased probability of graduating from high school) | 19 | 1. LGBTQ students perceive more support in their school when school staff, administrators, and teachers show support through actions and presence that take a stance against bigotry and defends marginalized students **(Blackburn, 2007; Liboro et al., 2015; Luecke, 2011; Mayberry et al., 2013)** 2. LGB youths who reported having natural mentors (teachers, staff members, school administrators) were about three times as likely to graduate from high school compared to those who do not have natural mentors **(Drevon et al., 2016)** 3. The more types of safe adults at school, the greater the school engagement of LGBTQ youth **(Mayo, 2013a; Seelman et al., 2012, 2015)** 4. Supportive school staff members were able to provide community partnerships for LGBT students for opportunities and resources access outside of school, increasing the sense of belonging with a community **(Liboro et al., 2015)** 5. Supportiveness of school staff was associated with increased intentions to seek help for suicidal thoughts and a positive school climate **(Colvin et al., 2019)**. 6. The importance of social work professionals as "safe" adults at school impacting academic engagement, and school connectedness and support **(Craig et al., 2018)**. 7. Teacher intervention were associated with higher levels of safety for LGBTQ youth **(De Pedro et al., 2018)**. 8. Supportive adults in schools were shown to be knowledgeable about SOGI issues and implement school and classroom procedures that help LGBTQ youth feel safe in their schools **(De Pedro et al., 2018)**. 9. Supportive teacher expectations for SM students resulted in these students having increased educational achievement **(Fenaughty et al., 2019)**. 10. From the perspective of TGNC children, they perceive that teachers have the power to foster a safe classroom climate and environment, supporting the transition of transgender youth **(Goldstein et al., 2018)**. 11. Supportive school staff allowed for students to create a community art gallery for students to foster awareness of LGBTQ issues in their school and surrounding environment **(Hall et al., 2018)**. 12. Teachers also mentioned becoming allies of LGBT students as a role model and not be bystanders to pejorative language, emphasizing the importance of behavioral management to establish a safe classroom space **(Pearce & Cumming-Potvin, 2017)** 13. Teachers were integral in opening conversations to be inclusive and relevant to all students to promote safety and acceptance in the classroom **(Shelton & Lester, 2018)**. 14. Teachers understood the importance of inclusive language use as an indicator of a supportive environment for trans/gender-diverse students in their classrooms **(Ullman, 2018)**. 15. Teachers, school psychologists, and principals are key stakeholders to develop skill sets to support LGBTQ+ students in fostering inclusion and acceptance **(Underhill, 2017)** 16. Positive teacher relationships suggests more positive wellbeing amongst all students, particularly in environments where there is heteronormative environments **(Vantieghem & Houtte, 2020)** |
| Having a high level of within-school adult support reduces negative outcomes (e.g. lower levels of sexual orientation-based victimization) | 10 | 1. Regardless of one's sexual orientation, student perceptions of greater adult support at school were consistently linked to their reports of lower levels of sexual-orientation victimization, school avoidance, and substance use, particularly for LG youths **(Darwich et al., 2012)** 2. School engagement predicts a decrease in fear-based truancy for those who have higher levels of fear at school. The presence of supportive adults at school reduced the number of days skipping school due to fear **(Mayo, 2013a; Seelman et al., 2012, 2015)** 3. Having an adult ally at school is associated with a decrease in fear-based truancy **(Seelman et al., 2012).** 4. LGB youths who had a school adult connection were less likely to use substance and decreased mental health risks (e.g., suicidality; **Seil et al., 2014).** 5. Principals agreed that there is a need for increased efforts towards reducing bullying/discrimination towards LGBTQ youth, setting the tone for their schools **(Boyland et al., 2018)**. 6. Supportiveness of school staff was associated with fewer depressive symptoms **(Colvin et al., 2019)**. 7. The importance of social work professionals as "safe" adults at school decreased substance use **(Craig et al., 2018)**. 8. For SM Chinese adolescents, having supportive teachers mediated the effect of their suicidal behavior **(Huang et al., 2018)** 9. Access to school-based health centers can ameliorate health disparities among SMY youth, providing an inclusive and affirmative environment to reduce SMY's barriers to health care **(Zhang et al., 2020)**. |
| Though teachers and school staff can be supportive adult figures to reduce negative psychological outcomes, have been shown to be ineffective in supporting LGBTQ students | 7 | 1. Students have reported that teachers sometimes do not know how to intervene effectively, with the two most common intervention strategies for verbal harassment include 1) stopping the harassment and 2) explaining why it is wrong. **(Hillard et al., 2014)** 2. Teachers were shown to not feel prepared to support LGBTQ students and needed more knowledge and information, to which collaborating with GSAs helped improve their pedagogy **(Luecke, 2011; Mayo, 2013a)** 3. Though there were benefits in identifying and having a supportive adult, 80.9% reported never talking to a teacher regarding SOGI topics, 70.8% never talked to a school/mental health counselor and 86.5% never talked to a school administrator about issues related to SOGI during high school **(Bidell, 2014)** 4. GSA students perceive faculty silence surrounding SOGI topiccs as a normative feature of the school environment and the lack of overt resistance to the existence of GSAs in their schools **(Mayberry et al., 2013)** 5. Within-school adult support was not as protective against suicidality compared to outside school adult support **(Coulter et al., 2017)**. 6. Teachers need to increase their responsibility to support students as transgender children were tired/burdened to educate others on how to support them **(Goldstein et al., 2018)**. |
| School staff perceived external support as key to ensure coordination to foster staff support for LGBTQ students | 4 | 1. School staff emphasized the importance of having a coordinator external to the school as this person was able to continuously push the school's administrators to be willing to talk about issues of sexuality with instant availability to provide support for curricular efforts, activities and adapting to student, staff, and school needs, subsequently reducing harassment for LGBTQ students **(Horowitz and Itzkowitz, 2011; Liboro et al., 2015; Luecke, 2011)** 2. Having an external source of support showed statistical improvements where students reported that LGBTQ students were accepted at their school 'sometimes' or 'a lot of the time'. **(Horowitz and Itzkowitz, 2011)** 3. External supports such as sexuality education workshops were shown to have had significant positive effects on teachers' beliefs and behaviors' to support SGM students **(Kwok, 2018)** |
| **School Policies** | | |
| The socio-political values of the wider community beyond the school have an impact on school policies and staff attitudes | 7 | 1. Students and faculty mentioned non-progressive attitudes and beliefs about LGBTQQ people due to political and religious conservatism led to faculty hesitation about forming a GSA and that the administration yielded to the beliefs and attitudes of the larger community regarding sexual orientation **(Lassiter and Sifford, 2015; Mayberry et al., 2013; Wright et al., 2012)** 2. GSA ban resulted in increased powerlessness, frustration, disappointment, and anger as school counselors believed that students are prevented from experiencing benefits of GSA presence and sent a message regarding the visibility of LGBTQQ community **(Lassiter and Sifford, 2015)** 3. Supportive staff members attempted to foster resistance through systematic inclusion by framing that the ban will foster risks as the school is not meeting the needs of the students and that the school should be altruistic to support all students (resistance needs to be framed as a general support and benefits for all students to reduce the hesitance) **(Lassiter and Sifford, 2015; Mayberry et al., 2013; St. John et al., 2014)** 4. Certain GSAs were limited in the activities and spaces (only activities deemed important and appropriate) they were allowed to hold to minimize the risk of external resistance from parents and community **(Lassiter and Sifford, 2015; Mayberry et al., 2013)** 5. School policies were sometimes more enforced unequally to LGBTQ youth as they were being punished for PDA and violating dress code. The lack of inclusive school policies, in combination with the lack of support from teachers, staff and administration led to students resisting to protect themselves **(Snapp et al., 2015b).** 6. Supportive government and school board policies allowed for GSAs in this region to flourish and foster community connections, showing an importance for the larger community to support schools **(Liboro et al., 2015; St. John et al., 2014)** 7. Local communities, regions, and districts result in variations in their school plan to support LGBTQ students (and what can and cannot be done in their policy work based on funding and backing) **(Fantus & Newman, 2021)** 8. Effecting change across multilevel contexts within schools requires a school-wide approach and communal investment from within and in the local community in order to promote the social, psychological, and physical safety for all students **(Fantus & Newman, 2021).** |
| Implementing inclusive and anti-discriminatory policies have been shown to be effective in fostering a safer school space for LGBTQ students, fostering both positive psychological outcomes and reducing social-emotional risks | 5 | 1. Schools with higher reported implementation of policies and best practices had lower levels of reported bullying or discrimination against LGBTQ students **(Boyland et al., 2018)**. 2. Effective bills such as Bill 13 were shown to allow LGBTQ youth to create a space for them to transform their own lives and offering opportunities of activism for marginalized youth **(Iskander & Shabtay, 2018)**\ 3. Inclusive policies allow for events such as Pride Prom and Day of Silence that provides a safer environment for gay and lesbian youth to celebrate their graduation **(Sutherland, 2019)** 4. Policy frameworks have shown that it is not only important to provide a safe context for students but more to challenge the hetero/cisnormative dynamic present in policy documents and classroom and create concrete action to create an empowering climate **(Ullman, 2018).** 5. Policies are needed to ensure collaboration across professionals to support legislation to recognize sexuality and gender issues in schools **(Kwok, 2018)** |
| School policy and community support showed challenges and mixed conclusions in fostering positive outcomes | 4 | 1. Higher proportions of students who reported inclusive school policies predicted lower perceptions of safety based on gender nonconformity **(Toomey et al., 2012)** 2. Bullying policies were not consistently enforced and was not significantly predicting LGBTQ safety and victimization **(Boyland et al., 2018; De Pedro et al., 2018).** 3. Community support was not related to decreased rates of harm **(Ross-Reed et al., 2019)** |
| **School Climate** | | |
| A positive school climate can reduce negative emotional-behavioral outcomes and reduce educational risks. | 4 | 1. For LGB and heterosexual students who are in a positive school climate and not experiencing homophobic teasing, they scored the lowest on depression/suicidality, alcohol/marijuana use, and truancy **(Birkett et al., 2009)** 2. Students who reported a more supportive and positive school environment were less likely to report anxiety or depressive symptoms and less suicidality **(Colvin et al., 2019; Denny et al., 2016)** 3. SM students who were more involved in school-based activities and had stronger school connectedness were less likely to have ever used alcohol/marijuana, drugs, and decreased physical fights **(Ethier et al., 2018)**. 4. Teacher reports of more supportive school environments were associated with fewer depressive symptoms among male but not female SM students **(Denny et al., 2016)** |
| A positive school climate can foster positive psychosocial and educational outcomes | 11 | 1. By fostering safer school climates by combining GSA-type clubs with other programs such as LGBT-affirming school-wide campaigns and significant events, this promoted tolerance, respect and inclusion for LGBT youths **(Liboro et al., 2015; Mayo, 2013a; Wernick et al., 2016)** 2. A positive school climate has shown to help teachers feel comfortable with advocating for LGBT students **(Luecke, 2011; Mayo, 2013a)** 3. Student school engagement was found to be a significant predictor of student GPA **(Seelman et al., 2012)** 4. LGBTQ affirming school climates in rural school communities are associated with more positive perceptions of safety for LGBTQ youth **(De Pedro et al., 2018)**. 5. SM students who were more involved in school-based activities, had supportive school structures, and had stronger school connectedness felt safer in schools and had increased achievement **(Ethier et al., 2018; Fenaughty et al., 2019)**. 6. Students, parents, and school staff mentioned the importance of having a safe space as a deciding factor to attend the school, not only having a safe space but a thriving space where students are recognized, accepted, and are able to be present and participate in their school **(Hope & Hall, 2018)** 7. Irrespective of gender, students felt more positive psychological wellbeing in less heteronormative schools **(Vantieghem & Houtte, 2020)** 8. Classroom environments that were inclusive allowed LGBTQ students to be more inclusive and increased opportunities to understand diversity and differences **(Shelton & Lester, 2018)**. |
| A whole-school effort (e.g., promoting LGBTQ+ student-led change initiatives) is required to foster a positive school climate and lead to various positive outcomes | 10 | 1. Interventions such as staff development and training, LGBTQ-inclusive curriculum, GSA-type clubs, and awareness events are necessary to foster a positive school climate which in turn fosters educational and social success **(Horowitz and Itzkowitz, 2011; Wernick et al., 2016; Woolley, 2012)** 2. By providing their LGBT students access to more resources by connecting them with LGBT-positive community partners, this can create more opportunities to carry out programs such as professional development, staff training, and curricular changes that maximized the potential of the students, teachers, administrators, and board trustees, to attain a whole-school system environment that fostered a positive school climate **(Liboro et al., 2015)**. 3. GSAs, faculty and staff support, education and training for the school community, appropriate mental health services, school-home-community connections, and inclusive curriculum supports for GLBT students are all aspects of support systems needed to meet the needs of GLBT youth and foster a sense of positive school climate **(Robertson, 2008; Woolley, 2012; Wright et al., 2012)** 4. A positive school climate was attempted by students in a GSA by actively taking action to address anti-LGBTQ bias, to provide education, and address the silences on LGBTQ issues through whole school efforts such as school events and classroom discussions **(Liboro et al., 2015; Wernick et al., 2016; Woolley, 2012)** 5. By having a supportive principal, this facilitated a positive whole-school approach to support a student (Jaden) feeling comfortable with herself going through her transition **(Luecke, 2011)** 6. School districts found it was important to be able to create school-wide approaches (e.g., inclusive policies, school staff knowledge development, safe/supportive school environment, healthy/supportive peer connections, access to affirming services, community and family engagement), such as large-scale programmes, to be able to ensure the support of LGBTQ/TGNC students in schools **(Goodrich & Barnard , 2019)** 7. A whole school approach is needed to promote TGD activism and support TGD students' wellbeing. This includes school staff can create a safer school environment, having professional development opportunities for staff to feel knowledgeable, inclusive policies, and having an inclusive curriculum **(Jones, 2017)**. 8. Students mentioned the importance of a school-wide approach to support LGBTQ students. This included inclusive language from educators, seeing supportive staff as role models, inclusive curriculum, inclusive policies (e.g. dress), and overall school and classroom climate being safe for LGBTQ students **(Palkki & Caldwell, 2018)** |
